# The Eps8/IRSp53/VASP Network Differentially Controls Actin Capping and Bundling in Filopodia Formation

**DOI:** 10.1371/journal.pcbi.1002088

**Published:** 2011-07-21

**Authors:** Federico Vaggi, Andrea Disanza, Francesca Milanesi, Pier Paolo Di Fiore, Elisabetta Menna, Michela Matteoli, Nir S. Gov, Giorgio Scita, Andrea Ciliberto

**Affiliations:** 1 IFOM Foundation, Institute FIRC of Molecular Oncology, Milan, Italy; 2 Microsoft Research-University of Trento Centre for Computational and Systems Biology (CoSBi), Povo (Trento), Italy; 3 Dipartimento di Medicina, Chirurgia ed Odontoiatria, Universita' degli Studi di Milano, Milan, Italy; 4 Dipartimento di Farmacologia, CNR Institute of Neuroscience, Center of Excellence on Neurodegenerative Diseases, Milan, Italy; 5 Department of Chemical Physics, Weizmann Institute of Science, Rehovot, Israel; Stanford University, United States of America

## Abstract

There is a body of literature that describes the geometry and the physics of filopodia using either stochastic models or partial differential equations and elasticity and coarse-grained theory. Comparatively, there is a paucity of models focusing on the regulation of the network of proteins that control the formation of different actin structures. Using a combination of *in-vivo* and *in-vitro* experiments together with a system of ordinary differential equations, we focused on a small number of well-characterized, interacting molecules involved in actin-dependent filopodia formation: the actin remodeler Eps8, whose capping and bundling activities are a function of its ligands, Abi-1 and IRSp53, respectively; VASP and Capping Protein (CP), which exert antagonistic functions in controlling filament elongation. The model emphasizes the essential role of complexes that contain the membrane deforming protein IRSp53, in the process of filopodia initiation. This model accurately accounted for all observations, including a seemingly paradoxical result whereby genetic removal of Eps8 reduced filopodia in HeLa, but increased them in hippocampal neurons, and generated quantitative predictions, which were experimentally verified. The model further permitted us to explain how filopodia are generated in different cellular contexts, depending on the dynamic interaction established by Eps8, IRSp53 and VASP with actin filaments, thus revealing an unexpected plasticity of the signaling network that governs the multifunctional activities of its components in the formation of filopodia.

## Author Summary

Cells move and interact with the environment by forming migratory structures composed of self organized polymers of actin. These protrusions can be flat and short surfaces, the lamellipodia, or adopt an elongated, finger-like shape called filopodia. In this article, we analyze the ‘computation’ performed by cells when they opt to form filopodia. We focus our attention on some initiators of filopodia that play an essential role due to their interaction with the cell membrane. We analyze the formation of these filopodia initiators in different genotypes, thus providing a way to rationalize the behaviors of different cells in terms of tendency to form filopodia. Our results, based on the combination of experimental and computational approaches, suggest that cells have developed molecular networks that are extremely flexible in their capability to follow the path leading to filopodia formation. In this sense the role of an element of the network, Eps8, is paradigmatic, as this protein can both induce or inhibit the formation of filopodia depending on the cellular context.

## Introduction

Filopodia, actin-rich, finger-like structures that protrude from the cell membrane of a variety of cell types, play important roles in cell migration, neurite outgrowth and wound healing [1]. Filopodia are characterized by a small number of long and parallel actin filaments that deform the cell membrane, giving rise to protrusions. In order for filaments to grow to the characteristic length observed in filopodia, capping proteins, specialized molecules that inhibit actin polymerization, need to be locally inhibited or sequestered and nucleation of new filaments needs to be favored. Furthermore, individual actin filaments are not sufficiently stiff to deform the cell membrane [2]. Proteins, such as VASP-family proteins are thought to be required to promote the initial transient association of actin filaments as they directly [3] or indirectly antagonize capping proteins [4], capture barbed ends [5] and cross-link actin filament [4], [5]. Furthermore, they can act as processive filament elongators especially upon high-density clustering, at least in vitro [4], [6], [7]. Actin filaments are then further stabilized by other crosslinkers, such as fascin, thus permitting the formation of bundles of sufficient stiffness to overcome buckling and membrane resilience [8]. Thus, in a simplified view, capping proteins can be seen as inhibitors, while bundling proteins are among the necessary components of filopodia formation. Consistently with this picture, removal of Capping Protein (CP) causes an increase in the number of filopodia [9]. Vice versa, cells devoid of the actin crosslinker fascin display a reduced amount of filopodia [8].

This simple rule does not seem to apply easily to the actin remodeler Eps8, which plays complex roles in filopodia formation reflecting its diverse biochemical functions. Eps8 can efficiently cap barbed ends when bound to Abi-1 [10], while it crosslinks actin filaments, particularly when it associates with IRSp53 (Insulin Receptor Tyrosine Kinase Substrate of 53 KD) [11], [12], [13], [14], a potent inducer of filopodia via its ability to bind actin filaments and deform the plasma membrane (PM) through its IMD domain [15]. Consistent with its dual function, the role of Eps8 in filopodia formation is cell context-dependent. In HeLa and other epithelial cell lines, the ectopic expression of Eps8 in the presence of IRSp53 promotes the formation of filopodia, while its removal reduces them [12]. The opposite behavior is observed in primary hippocampal neurons, where genetic removal of Eps8 increases the formation of axonal filopodia [16].

In order to rationalize the information described so far, we propose that the process of filopodia formation proceeds in a step-wise fashion. During an initial phase, multiple and simultaneous binding reactions (primarily involving cappers, bundlers and filamentous actin) lead to the formation of pre-existing filaments into bundles. In a second phase, elongation of these bundled filaments is required to support the extension of filopodia. Hitherto, efforts in modeling filopodia formation have focused on the structure and physical properties of filopodia [17], [18], [19], [20] as well as into the role of specific proteins in modulating the characteristic of individual filopodia [21]. More recently, some models have started to couple a detailed biophysical description of filopodia dynamics with some of the molecules involved with capping and bundling [22], [23]. However, it is extremely challenging to treat with the same model filopodia formation in terms of theory of elasticity or stochastic simulations while keeping track of the full behavior of the complex protein-protein interactions underlying the formation of bundled filaments. Particularly, the effect of modifications (e.g. gene deletions or over-expression) affecting the network has never been approached so far with computational methods.

Here, we combined computational models, *in-vitro* and *in-vivo* experiments and describe in mathematical terms the behavior of the protein-protein interaction network underlying the formation of bundled filaments using a minimal but biologically relevant module, centered on the IRSp53/Eps8/VASP pathway, with the aim of defining general principles governing the formation of filopodia in different cellular contexts.

## Results

### The IRSp53/Eps8/VASP molecular network

**Figure 1:**
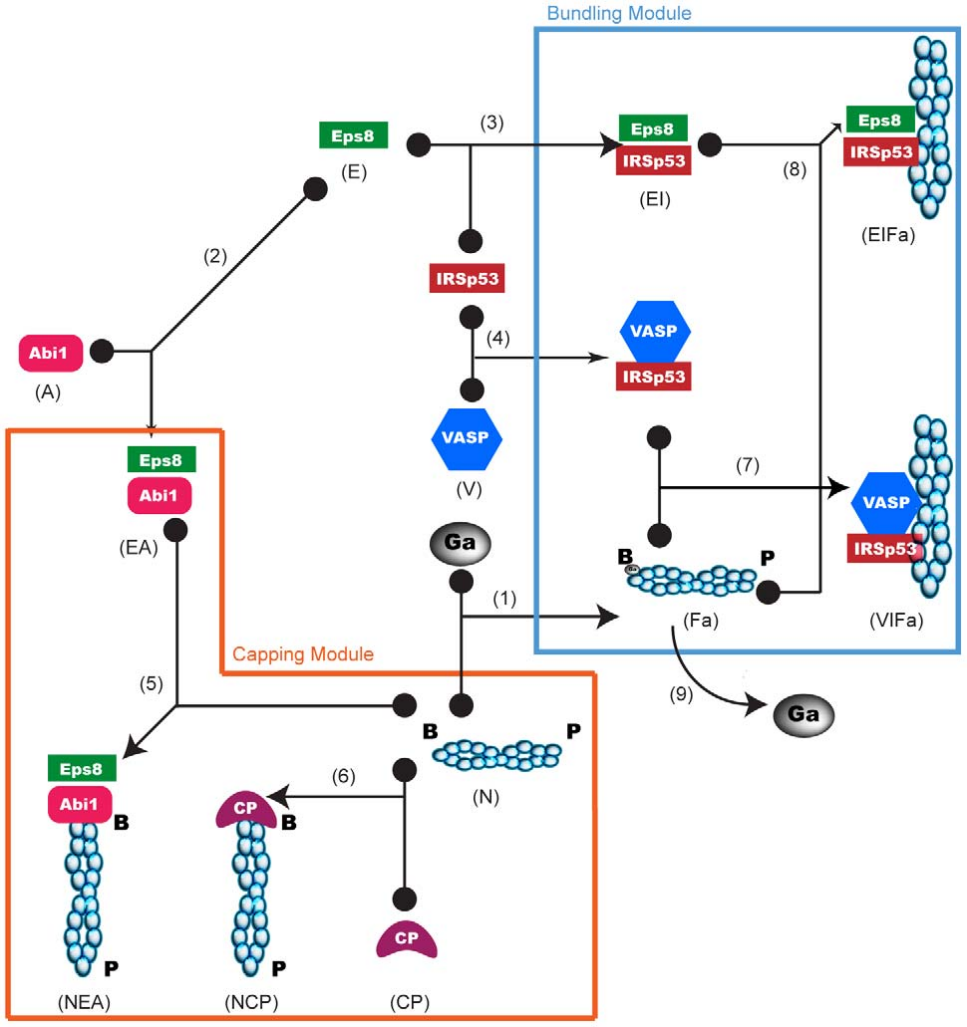
Eps8 and IRSp53 effector network. Network showing the main interactors of Eps8 and IRSp53 involved in the regulation of filopodia formation. Black filled dots indicate substrates of a reversible binding reaction, whose product is pointed by an arrow. Turnover of filamentous actin (reaction (9)) is the only irreversible reaction depicted in the diagram. In the network, we identify two different modules, a capping module, which includes the binding reactions between cappers and barbed ends, and a bundling module, which includes the binding reactions between bundlers and filamentous actin. CP represents Capping Protein, cyan circles are polymerized monomers of actin; the red circle marked Ga is G-actin. B and P mark the barbed and pointed ends, respectively, of a filament of actin. Reaction numbers and the shortened names in parentheses under the icons allow an easy interpreation of the equations of the model (Table 1 in Text S1).

In this section, we first introduce the topology of the network underlying filament bundling (Fig. 1). Together with the network that we intend to model, we also enlist the assumptions adopted to translate the network in mathematical formalism. We then discuss the determination of the parameters used for the simulations.

### Polymerization of actin

*In-vivo*, capping proteins block most of the barbed ends preventing uncontrolled filament elongation [24]. Additionally, most of the G-actin available for polymerization is bound to profilin, a monomeric actin binding protein that promotes the exchange of ADP to ATP and decreases the affinity of monomeric actin for filament pointed ends and spontaneous filament nucleation [25]. Accordingly, in our model, polymerization occurs at barbed ends only (equations in Table 1 in Text S1). Under these conditions, the rate of polymerization is proportional to the total G-actin concentration and to the number of free barbed ends. While local G-actin concentration can vary due to local polymerization and depolymerization fluxes [26], [27], the total concentration of G-actin in cells is maintained buffered through mechanisms involving ATP turnover and actin sequestering proteins [28], and thus we treat it as a fixed parameter in our model. This choice is particularly suited to our analysis, which aims to reproduce steady state behaviors and not transient dynamics. We used a concentration of 10 µM of G-actin available for polymerization in cells as estimated in [27], [29].

As for depolymerization, we introduce dissociation of monomers from barbed ends. Since for our purposes a simplified description of actin polymerization suffices, we ignore pointed ends dynamics, while, following a formalism presented in [30] we include a turnover for actin proportional to the total amount of F-actin. Notably, even if we explicitly account for pointed ends polymerization and depolymerization together with a variable amount of G-actin, the results of the model are qualitatively similar (unpublished results). Finally, since the model is based on ordinary differential equations, we do not explicitly take into account individual filaments with variable amounts of actin, but identify a bulk of polymerized actin, F-actin (Fa).

### Capping

In the cell types we examine, two cappers, CP and the Eps8:Abi-1 complex, play important roles in filopodia formation. We thus explicitly introduce these two molecular species and their interaction with barbed ends in the network (Fig. 1).

Cells tightly control polymerization by maintaining most barbed ends capped, since uncapped filaments in cellular extracts would elongate due to G-actin concentrations higher than the critical concentration for barbed ends [31]. Thus, in our model we assume that, at the steady state, the nucleation and depolymerization of filaments results in a fixed total number of barbed ends and that the concentration of capping proteins (CP and the complex Eps8:Abi1) is sufficiently high to cap most of them.

The behavior of the system “out of steady state” (e.g., bursts of polymerization giving rise to the growth of individual filopodia) is not analyzed experimentally and thus, as anticipated, will not be reproduced by the simulations. We use the model only to reproduce changes in the steady state behavior of the network in various genetics backgrounds where components of the network are either deleted or over-expressed. Finally, we purposely avoided including the anti-capping activity of VASP family members as its role in filopodia formation is still unclear [32], and little is known as to whether this activity is regulated upon binding of these proteins to IRSp53.

### Bundling complexes

#### Bundling activity of EPS8:IRSp53

**Figure 2:**
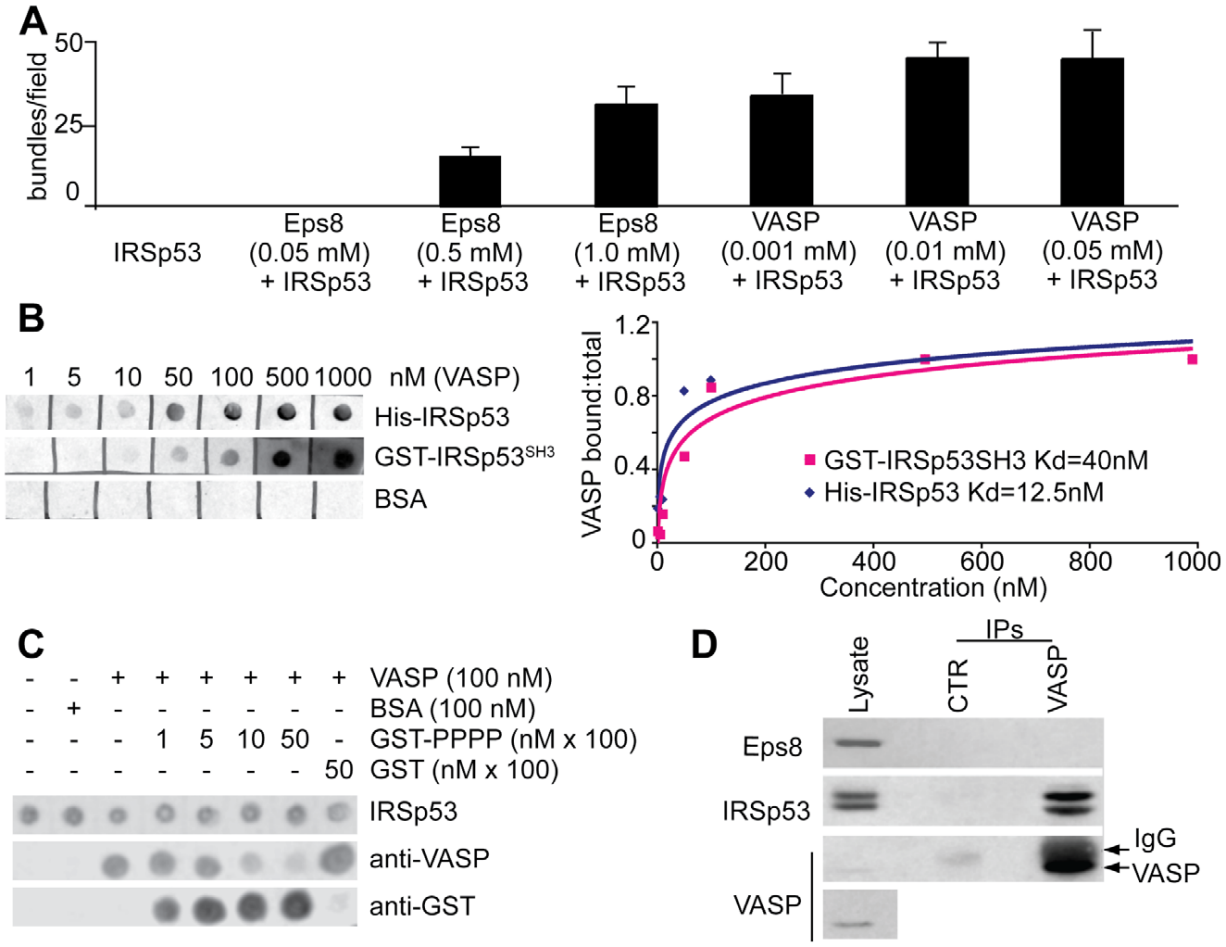
VASP synergizes with IRSp53 in bundling actin filaments and competes with Eps8 for IRSp53 binding. **a.** Isolated VASP and Eps8 bundle actin filaments with low efficiency, which is enhanced by their association with IRSp53. The bundling efficiency was determined by measuring the number of bundles/field obtained in fluorescence microscopy-based F-actin-bundling assays as described and shown in Fig. S1A–B. At least 10 fields per experiment performed in triplicates were scored. Data are the mean ± s.e.d. **b.** Measurement of IRSp53 and VASP interaction. Equal amounts (10 pmoles) of His-IRSp53, GST-IRSp53-SH3 or BSA were spotted onto nitrocellulose and incubated with increasing concentrations of purified VASP. The nitrocellulose filter was then subjected to WB analysis using anti-VASP antibody (Ab). The fraction of VASP bound was plotted against the concentrations of total VASP. An apparent dissociation constant was calculated using standard procedure as described in [12]. **c.** The proline rich region of Eps8 (PPP) competes with VASP for binding to IRSp53. Equal amounts (10 pmoles) of His-IRSp53 spotted onto nitrocellulose and incubated with purified 100 nM VASP or BSA as control, in the absence or the presence of increasing amounts of the proline-rich region of Eps8 (GST-PPP) or GST. The filters were immunoblotted with the indicated abs. **d.** VASP forms a complex with IRSp53 *in-vivo*. Lysates (1 mg) of HeLa cells were immunoprecipitated with anti-VASP or with control abs. Lysates (20 µg) and immunoprecipitates (IP) were immunoblotted with the indicated abs. The bottom panel is a longer exposure to visualize endogenous levels of VASP.

The Eps8:IRSp53 complex was previously characterized as an actin bundler capable of inducing filopodia formation [12]. Individually, Eps8 and IRSp53 are both weak bundlers, but they can interact forming an Eps8:IRSp53 complex that displays increased actin bundling activity in the bulk solution (Fig. 2A and Fig. S1A–B). In the model, the complex Eps8:IRSp53 favors bundling by binding to the side of actin filaments, thus generating a “filopodia initiation complex” (i.e., Eps8:IRSp53:Fa) (see later for a thorough explanation). This reaction, as all others, takes place following simple mass action kinetics.

Thus, Eps8 can form complexes with Abi-1, capable of capping activity, and with IRSp53, capable of bundling. While Abi-1 and IRSp53 bind to Eps8 *in-vitro* on different surfaces [12], *in-vivo*, Eps8 is present in two distinct sub-populations: together with Abi-1 on barbed ends along the cell membrane and together with IRSp53 along bundled actin filaments such as microspikes and filopodia, suggesting the presence of two distinct complexes. Consistently, co-immunoprecipitation experiments of endogenous proteins in various cell lines showed no evidence of the existence of the triple complex [16]. Thus, in our model Eps8 can bind IRSp53 or Abi-1; the two binding reactions being in competition with each other.

#### Bundling activity of VASP

The ability of VASP to favor filopodia formation is well established, but the biochemical mechanisms through which this function is exerted are still controversial due to a variety of activities that this protein possesses [4], [5], [7], [32], [33], [34], [35]. While VASP displays actin bundling ability in *in-vitro* bulk experiments (Fig. 2A and S1A–B), this does not reflect in the capability to induce filopodia formation when expressed alone *in-vivo* (Fig. S1C). This result prompted us to seek possible factors that cooperate with or directly enhance VASP crosslinking activity.

One candidate protein that may fulfill this latter role is IRSp53. Binding of IRSp53 to Mena, a member of the VASP-family proteins, has been previously reported [36]. Moreover, the two proteins were shown to act in synergy in promoting filopodia formation supporting their functional interaction [36]. In keeping with this latter notion, functional interference with VASP-family proteins by sequestering away from the plasma membrane in cells over-expressing IRSp53 decreases the number of filopodia, hinting that VASP might act downstream of IRSp53 [12]. Intrigued by this possibility, we tested for synergies between VASP and IRSp53 in bundling actin filaments. In the presence of excess IRSp53, the ability of VASP to bundle filaments in *in-vitro* bulk experiments was increased 100 fold (Fig. 2A and S1A–B). This was paralleled by the ability of IRSp53 to localize VASP at sites of membrane curvature and to cause formation of filopodia *in-vivo* (Fig. S1C), similar to the Eps8:IRSp53 complex. To further characterize the interaction between VASP and IRSp53, we employed purified proteins and *in-vitro* assays. VASP binds to IRSp53 with significant affinity in the nM range (Fig. 2B), mainly through an interaction between the proline rich region of VASP and the SH3 domain of IRSp53. This latter domain also mediates the binding to Eps8, suggesting that VASP and Eps8 may directly compete for binding to IRSp53's SH3 domain. Notably, the affinity between Eps8 and IRSp53 is very similar to the affinity between VASP and IRSp53 (dissociation constants k_D_EI_ = 10 nM and k_D_VI_ = 12.5 nM, respectively) [12].

We thus set out to test directly whether Eps8 and VASP can compete for IRSp53 binding both *in-vitro* and *in-vivo*. Addition of the proline-rich region of Eps8 (PPP), the minimal region of interaction with the SH3 domain of IRSp53, to a fixed amount of VASP and IRSp53 decreased the amount of VASP:IRSp53 complex formed in a concentration-dependent manner (Fig. 2C). Additionally, *in-vivo*, IRSp53, but not Eps8, could be recovered on anti-VASP immunoprecipitates of HeLa cell extracts suggesting the existence of two distinct, mutually exclusive complexes (Fig. 2D).

Based on this evidence, we introduced in the model a second interactor of IRSp53, VASP, that competes with and is able to cause filopodia formation independently of Eps8 (Fig. 1). We estimated the affinity of the Eps8:IRSp53 complex for the side of the actin filament from low-speed centrifugation assays using the bundling domain of Eps8, and from similar experiments measuring the affinity of the IMD domain of IRSp53 [10], [37]. The affinity of VASP:IRSp53 for the actin filament was assumed to be 100 fold higher based on its ability to induce actin crosslinking at lower concentrations (Fig. 2A and Fig. S1) (notably, an increase of 1000 times, closer to the experimental value, would not change the result). Although these affinities are deduced from bulk experiments, we assume that they remain roughly unchanged even when the “filopodia initiation complexes” are formed at the PM. Likewise Eps8:IRSp53, we introduce binding of VASP:IRSp53 to filamentous actin following simple mass action kinetics, to form a “filopodia initiation complex”, VASP:IRSp53:Fa.

### Filopodia formation in the model

The formation of filopodia requires a number of other components in addition to those included in the model, most importantly fascin. However, we argue that the filopodia initiation complexes Eps8:IRSp53:Fa and VASP:IRSp53:Fa play a critical role likely in the initial phase of filopodia formation when filaments must be congregated in close proximity to the plasma membrane. These two filopodia initiation complexes share the critical and unique property to be anchored, primarily through IRSp53 and its membrane curvature sensing IMD module, to the plasma membrane, and thus show a high affinity for convex membrane curvature [1], [15]. Under these conditions, we hypothesize that the two complexes are ideally located to facilitate the “convergence” of actin filaments by promoting their bundling at the PM-oriented barbed ends. Notably and consistently with our hypothesis, actin filaments bundles have been recently proposed to be necessary for efficient protrusion by filling the space and providing mechanical support to the initial membrane deformation induced by IRSp53 that precedes the extension of filopodia [38]. Based on these considerations, we propose the “initiation of bundling” at the PM as the critical step in filopodia initiation, which is primarily due to the activity of Eps8:IRSp53 and VASP:IRSp53 and their ability to form initiation complexes with F-actin, upon which we focus our attention. Further supporting the important role of IRSp53-complexes in filopodia formation, theoretical studies show that membrane-bound protein complexes that have convex curvature and enhance actin polymerization, are able to initiate membrane protrusions [39]. As such, in our model we limit our analysis to the formation of Eps8:IRSp53:Fa and VASP:IRSp53:Fa, from now on abbreviated as FIC for “filopodia initiation complexes”.

**Figure 3:**
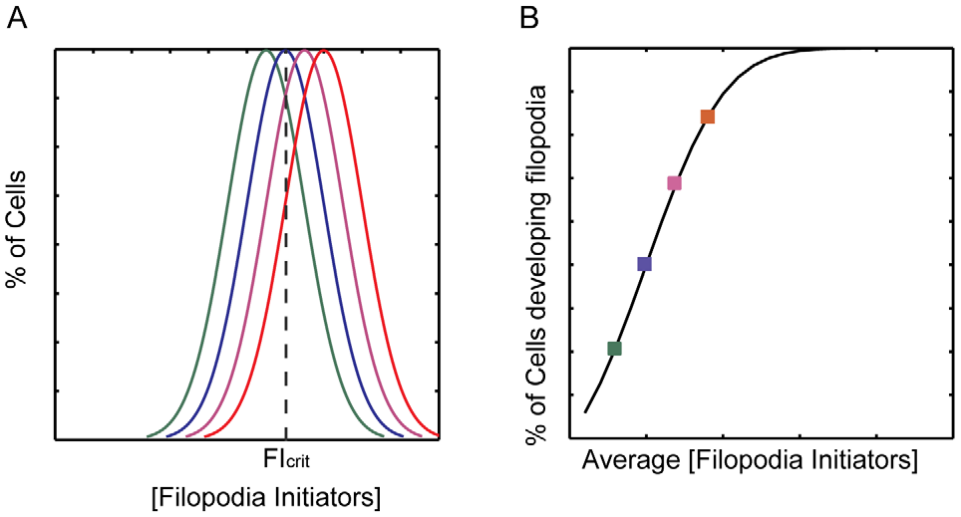
Average concentrations of filopodia initiator correlates with the probability of forming filopodia. **a.** Distributions of the concentration of filopodia initiators (FI) in cell populations with different mean values (μ) and identical standard deviationσ, computed as 
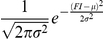
. The concentration of FI required for initiating the positive feedback loop (FI_crit_) is shown as a dotted line. As μ increases (different colored curves) the fraction of cells with FI>FI_crit_ increases. **b.** Fraction of cells in a population with FI>FI_crit_ as a function of the average FI concentration μ. Different color squares represent the fraction of cells for the different Gaussians shown in A. To calculate the amount of cells with FI>FI_crit_, we simply integrate the Gaussian from FI_crit_ to infinite, 
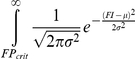
.

In a given cell population, the concentrations of the two FIC are expected to be distributed according to a normal (Gaussian) distribution centered around a mean value. Notably, only some of the cells of a population will develop filopodia, whereas others will not, accounting for the observation that filopodia formation shows a threshold behavior [40]. Recent models [39], [41] allow us to rationalize the threshold behavior based on a positive feedback loop triggered by FIC localized at the plasma membrane. When the mean concentration of FIC increases over a threshold value, they induce the spontaneous initiation of membrane protrusions through the following positive feedback mechanism: a local higher concentration of initiation complexes induces a higher local actin polymerization and protrusive force, which creates a local membrane protrusion and drives the accumulation of even more complexes since they are attracted to the convex curvature at the protrusion tip. Filament elongation and anti-capping activities might also involved in this second step following the formation of FIC. Importantly, as explained above, both the FIC considered here belong to the class potentially involved in the loop, i.e. they have both convex curvature (IRSp53) and promote actin polymerization against the plasma membrane, by increasing filament stiffness through their bundling activity. Accordingly, we hypothesize, following this model, that only the fraction of cells that reaches the threshold value of initiators concentration can activate the feedback loop and develop filopodia, as shown for a generic system in Fig. 3A.

We can compute the fraction of cells that crosses the threshold for filopodia formation as a function of the mean value of FIC in the cell population, assuming that this latter has a normal distribution of FIC. The resulting fraction of cells developing filopodia has an Error-function (Erf) dependence on the average concentration; it increases linearly as the average concentration increases around the threshold value, and saturates far above or below (Fig. 3B). This result suggests that there is a regime where the average concentration of FIC is linearly proportional to the fraction of cells that develop filopodia. Following this line of reasoning, we focused on a deterministic model that computes the average amount of initiation complexes present in the different genotypes.

To compare filopodia formation among different cell types, rather than measuring the percentage of cells that develop filopodia in a given genotype we normalized their value relative to the wild type (WT). The resulting ‘relative filopodia index’ (*RFI*), is the fraction of cells forming filopodia at steady state in a population of cells functionally interfered for the gene of interest (e.g., X), divided by the fraction of filopodia forming cells transfected with scrambled RNAi oligo:
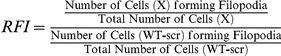
Accordingly, in the model we did not simply calculate the concentrations of filopodia initiation complexes, but a ‘filopodia initiation index’ (*FII*) defined as the concentrations of filopodia initiation complexes Eps8:IRSp53:Fa and VASP:IRSp53:Fa, normalized by their concentration in wild type cells:
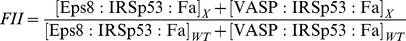
Throughout the manuscript, we will compare these two quantities to test the capability of the model to reproduce experimental data and predict new results.

### Cellular concentration of proteins and rate constants

To perform numerical simulations of filopodia formation in HeLa cells and neurons, we need to know the concentrations of the different species. Previous measurements showed that Eps8 and Abi-1 are present in similar concentrations in the two cell types, while Abi-2 is less concentrated in HeLa cells [16]. We then determined IRSp53 concentration through quantitative immunoblotting, and found that it is expressed at similar concentrations in both cell lines (Fig. S2A). As for VASP, we measured its concentration in HeLa cells to be in the submicromolar range (Fig. S2B). We could not directly measure the concentration of VASP in neurons due to the lack of antibodies equally effective against the mouse and human protein. However, reports in the literature show that Mena and EVL, the other two proteins in the VASP-family, are specifically expressed in brain at micromolar concentrations and that the three members of the family show high and overlapping expression levels in developing brain [42], [43], [44], [45]. Accordingly, we used a concentration of VASP-family protein higher in neurons than in HeLa cells. As for kinetic parameters for the various binding reactions, they were derived from the literature or measured directly (Fig. 2, Fig. S2 and Table 2 in Text S1).

Finally, when a protein was over-expressed, we assumed its concentration was increased 10 fold over its wild type values. For knockdown experiments via RNAi, we assumed that the protein concentration was reduced to 1/10^th^.

### Simulations and experimental results

After setting the topology of the network, having established the values of key parameters and identified an output that can be compared with the formation of filopodia, we utilized our model to explain the fundamental observation that removing Eps8 decreases filopodia formation in HeLa cells, but causes an increase in filopodia formation in neurons.

### Phenotypes of HeLa cells

**Figure 4:**
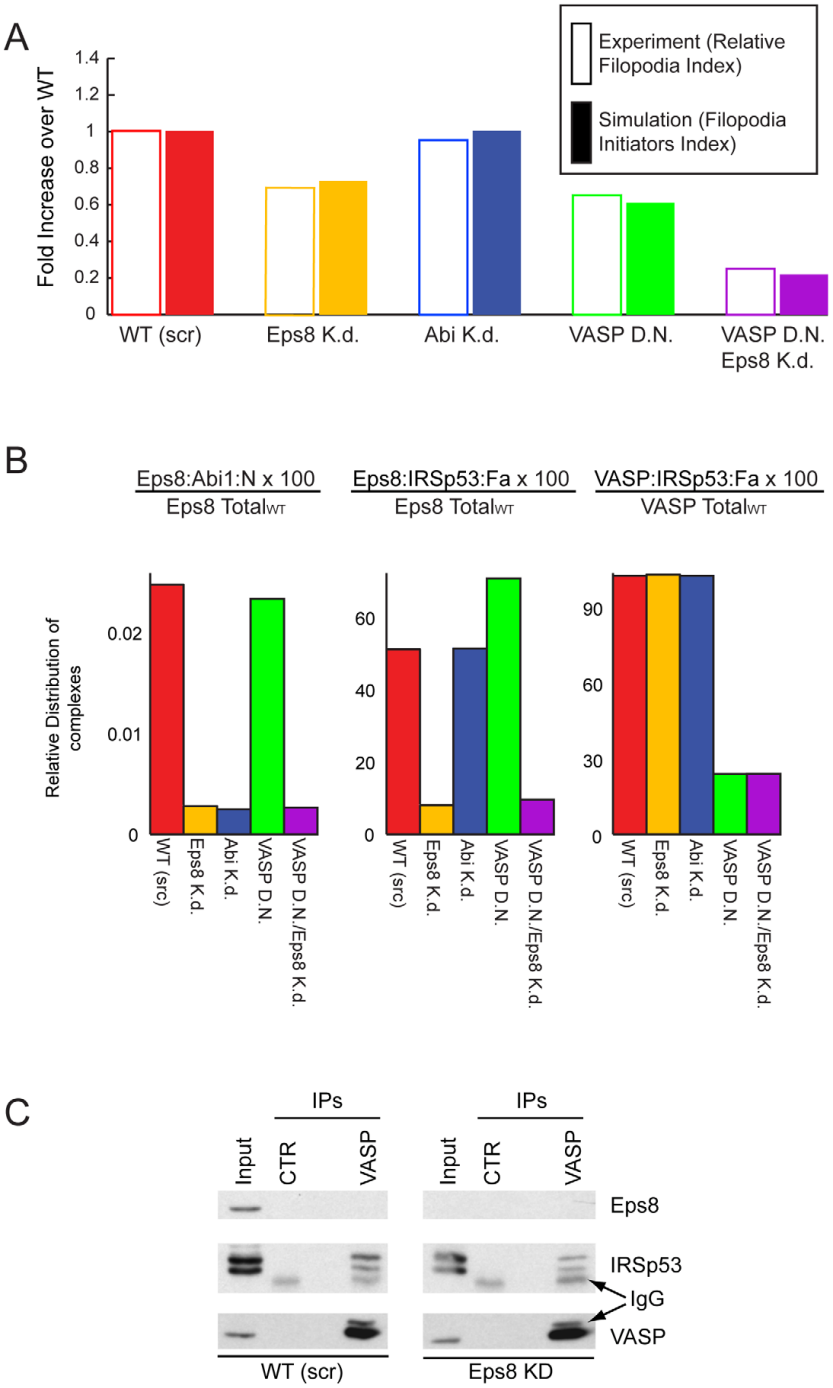
Eps8 plays a major role as a bundler, and not as a capper, in HeLa cells. **a.** Change in *RFI* and *FII* in the various genetic backgrounds. Empty rectangles represent experimental results (see Table 3 in Text S1), filled rectangles simulations of equations in Table 1 in Text S1 and parameters in Table 2 and Table 3 in Text S1. **b.** Complexes formed in HeLa cells by Abi1, Eps8, IRSp53, and VASP in different genetic backgrounds, plotted as percentage of total protein concentration in the wild type. Simulations performed as in **a**. **c.** Removal of Eps8 from HeLa cells does not significantly increase the amount of VASP bound to IRSp53. Lysates (1 mg) of HeLa control cells treated with a scrambled oligo [WT (scr)] or interfered for Eps8 (Eps8 K.d.) were immunoprecipitated with VASP or control abs. Lysates (40 µg) and immunoprecipitates (IPs) were immunoblotted with the indicated abs. IgG are also indicated.

In HeLa cells, genetic experiments measuring filopodia formation were done under conditions of IRSp53 over-expression (a condition that we define as WT), which in the model translates with concentrations of IRSp53 10 times larger than concentrations of Eps8 and VASP. The *RFI* was then measured in wild type and in cells in which we individually knocked down Eps8 or Abi-1 or functional interfered with VASP proteins or both with Eps8 and VASP simultaneously [12]. We then compared the fold increase in *RFI* measured in these cells with the fold increase of the *FII* in the model and found a good agreement (Fig. 4A). According to our model, in HeLa cells over-expressing IRSp53 the majority of Eps8 is bound to IRSp53 and filamentous actin, and very little is capping barbed ends (compare the red bar in the first two panels of Fig. 4B). Similarly, in HeLa cells no Abi-1 or Abi-2 co-immunoprecipitated with Eps8 [16]. We used the model to have an inside view of what happens to filopodia initiators and other protein complexes in the various genetic mutants after RNAi interference of the individual proteins of the network.

Simulations show that Eps8 knock down caused a reduction in the amount of Eps8:IRSp53:Fa (Fig. 4B, compare red and orange bars in the second panel) leading to a decrease in the total amount of filopodia initiation complexes (Fig. 4A). Although VASP and Eps8 compete for the binding with IRSp53, in our model removal of Eps8 did not significantly increase the amount of VASP-family proteins bound to it (see IRSp53:VASP:Fa, where “VASP” includes VASP-family proteins, in Fig. 4B, red and orange bars in the third panel). We confirmed this prediction by immuno-precipitating VASP in WT HeLa and HeLa cells knocked down for Eps8 (Fig. 4C) and verifying that the amount of IRSp53 bound to VASP remained constant. Simulations suggest that VASP's role is very similar to that of Eps8: indeed, functional removal of VASP caused a decrease in VASP:IRSp53:Fa (Fig. 4B, red and green bars in the third panel) and filopodia initiators in general (Fig. 4A). As VASP and Eps8 are redundant activators of IRSp53, the simultaneous down-regulation of both causes an increased reduction in filopodia formation, as predicted by the model (Fig. 4A).

As for the capping activity of Eps8 in this cell line, our simulations suggest that it does not play an important role. The complex Eps8:Abi-1 is very scarce and the removal of Abi-1 did not affect the amount of filopodia initiators (Fig. 4A and red and blue bars in the second and third panels in Fig. 4B).

Thus, our model supports the idea that the primary capping protein in HeLa cells is CP, and that Eps8 acts almost exclusively as a bundling protein downstream of IRSp53.

### Phenotypes of hippocampal-neurons

**Figure 5:**
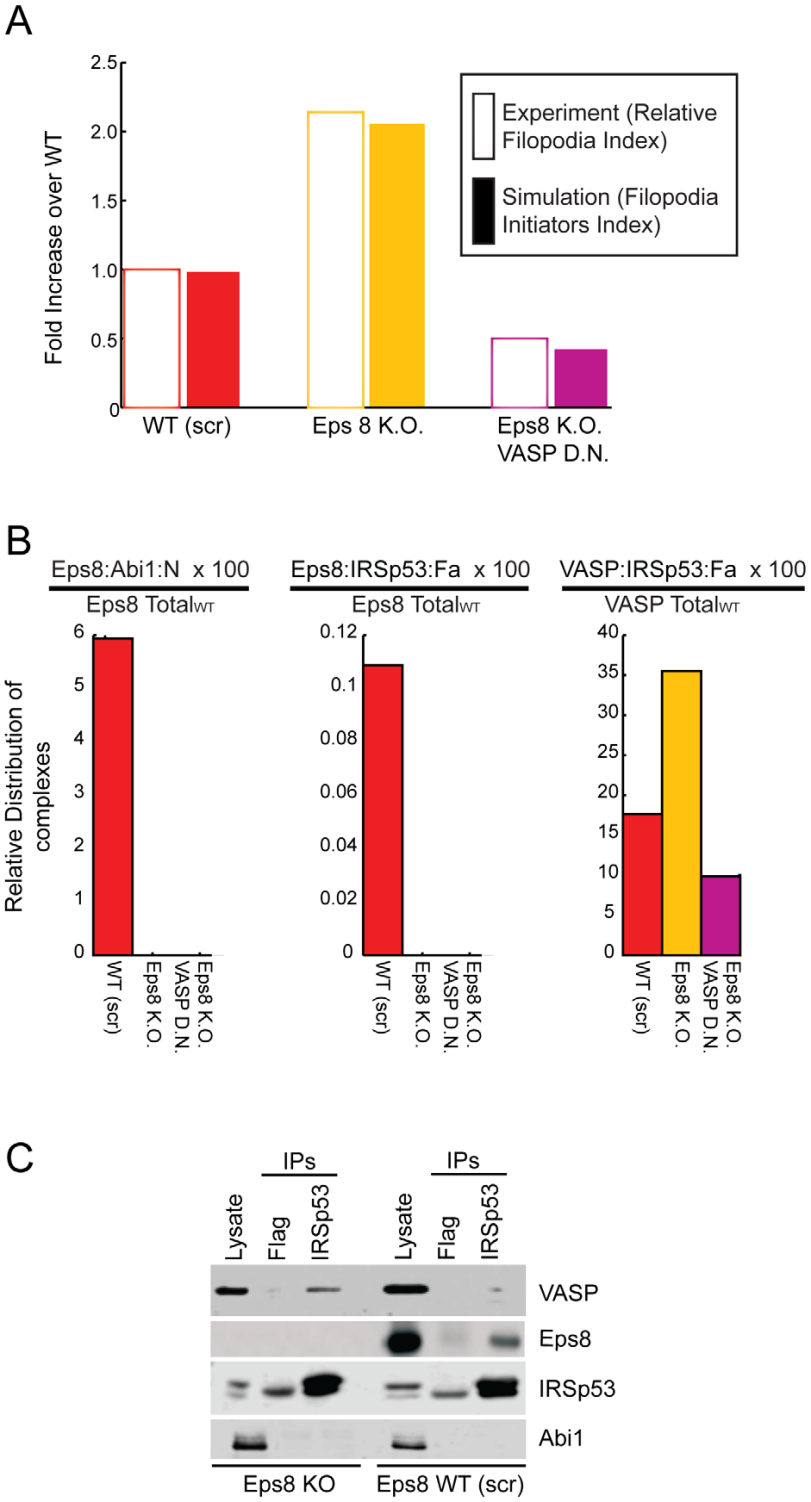
In Neurons Eps8 prevalently acts as a capper. **a.** Change in *RFI* and *FII* (i.e., Eps8:IRSp53:Fa and VASP:IRSp53:Fa normalized with respect to their concentrations in wild type cells) in the various genetic backgrounds. Empty rectangles represent experimental results (see Table 3 in Text S1), filled rectangles reproduce simulations of equations in Table 1 in Text S1 and parameters in Table 2 and Table 3 in Text S1. **b.** Complexes formed in HeLa cells by Abi1, Eps8, IRSp53, and VASP in the different genetic backgrounds plotted as percentage of total protein concentration in the wild type. Simulations performed as in **a**. **c.** Removal of Eps8 from neurons significantly increases the amount of VASP bound to IRSp53. Cortex and hippocampus lysates (1 mg) derived from Eps8 WT or KO mice were immunoprecipitated with anti-IRSp53 or anti Flag as control. Lysates (20 µg) and immunoprecipitates (IPs) were immunoblotted with the indicated abs.

In hippocampal neurons, removal of the different activators of IRSp53 leads to drastically different effects [16]. Functional interference with all VASP-family proteins inhibits filopodia formation similarly to what observed in HeLa cells after simultaneous ablation of Eps8 and VASP [12], [46] (Fig. 5A, red and purple bars). However, in neurons, but not in HeLa cells, the removal of Eps8 alone causes a large increase in the formation of filopodia along the neuronal shaft (Fig. 5A, red and yellow bars) [16], [46]. We used our model to understand the reasons behind this apparently paradoxical behavior.

The network described in Fig. 1 applies to both HeLa and hippocampal neurons; therefore we used the same set of equations and parameters for both cell types, with the noticeable exception of the concentrations of some proteins, Table 2 in Text S1. In hippocampal neurons, in fact, Abi-2 is expressed at much higher levels than in HeLa [16]. Similarly, all members of the VASP-family proteins are specifically and abundantly expressed in neurons and are presumably in excess with respect to IRSp53 as explained above. Moreover, at variance with respect to the experiments performed in HeLa, the analysis of axonal filopodia was conducted under conditions in which IRSp53 was not ectopically elevated. Accordingly, for neurons in the model we used a value of IRSp53 10 times smaller than in HeLa cells, and Abi-1 (which accounts for the presence of Abi-2) and VASP (which accounts for all VASP family members) were increased by a factor 5 (see Table 3 in Text S1).

The fold change in FIC derived from the simulations of our model were consistent with the experimental results obtained in WT, and Eps8 null hippocampal neurons either in the absence or the presence of a VASP dominant negative, which impairs the functional activity of all VASP family members [16] (Fig. 5A). A deeper analysis of the model's behavior allowed us to rationalize the phenotypes in molecular terms. Simulations of WT hippocampal neurons under condition of limiting IRSp53 (i.e. endogenous levels of the protein) suggest that a significantly higher fraction of Eps8 is bound to Abi-1 or Abi-2 compared to HeLa cells, to form the capping-active Eps8:Abi-1/2 complexes (compare red bar of the first panel in Fig. 4B with red bar of the first panel in Fig. 5B). Consistent with this notion, we previously reported that Eps8 binds a significant amount of Abi-1 and Abi-2 in neurons but not in HeLa cells [16]. Since a minimal fraction of Eps8:IRSp53 is bound to filamentous actin, the major filopodia initiator in neurons consists of VASP-family proteins bound to IRSp53 and Fa (compare red bars of panels two and three in Fig. 5B). Having defined the WT condition in hippocampal neurons, we set to analyze the change in steady state caused by the removal of Eps8.

In our simulations, removal of Eps8 increases the total amount of uncapped ends, causing an increase in the amount of filamentous actin (not shown). Moreover, we also observe an increase in the formation of the VASP:IRSp53 complex, due to the competition between VASP-family proteins and Eps8 for the scarce amount of IRSp53 available. As VASP:IRSp53 binds to filamentous actin with higher affinity than Eps8:IRSp53, the model predicts an increase in initiator complexes (compare red and orange bars in the third panel of Fig. 5B), which gives rise to a fold change in FIC for Eps8 knock out similar to what experimentally observed (Fig. 5A). We confirmed this result by immunoprecipitating IRSp53 in WT and Eps8 knock out neurons and observed that a higher amount of VASP was recovered in the knock out neurons (Fig. 5C). In our model, the increase in filopodia initiators due to Eps8 removal is reversed by the simultaneous functional interference with VASP-family proteins (Fig. 5A and orange and purple bars in panel four of Fig. 5B) consistent with what was experimentally measured [16].

We conclude that the role of Eps8 in neurons is more complex than in HeLa cells: in the former cells, it contributes to capping and competes with VASP-family proteins for the formation of filopodia initiators.

### Model's predictions

To further validate the model, we used it to make quantitative predictions about novel phenotypes. CP removal has been reported to cause an increase in filopodia formation in multiple cell-lines with high quantities of VASP-family proteins [9], but not in cell lines genetically devoid of VASP. The lack of filopodia formation in these latter cells was interpreted as an indication that VASP-family proteins are required for filopodia formation following the removal of capping proteins. This interpretation is in agreement with our model, according to which VASP induces filopodia formation via the initiator VASP:IRSp53:Fa. Our experiments also support this view, as we showed that VASP in complex with IRSp53 can induce filopodia formation *in-vivo* and formation of actin bundles *in-vitro*. However, in our model, VASP is not the only source of filopodia initiators. Eps8:IRSp53:Fa is also capable of inducing filopodia formation independently of VASP. Thus, we reasoned that in a setting where VASP cannot contribute to filopodia formation, CP removal should still lead to an increase in the fraction of cells producing filopodia via the parallel pathway provided by Eps8:IRSp53.

**Figure 6:**
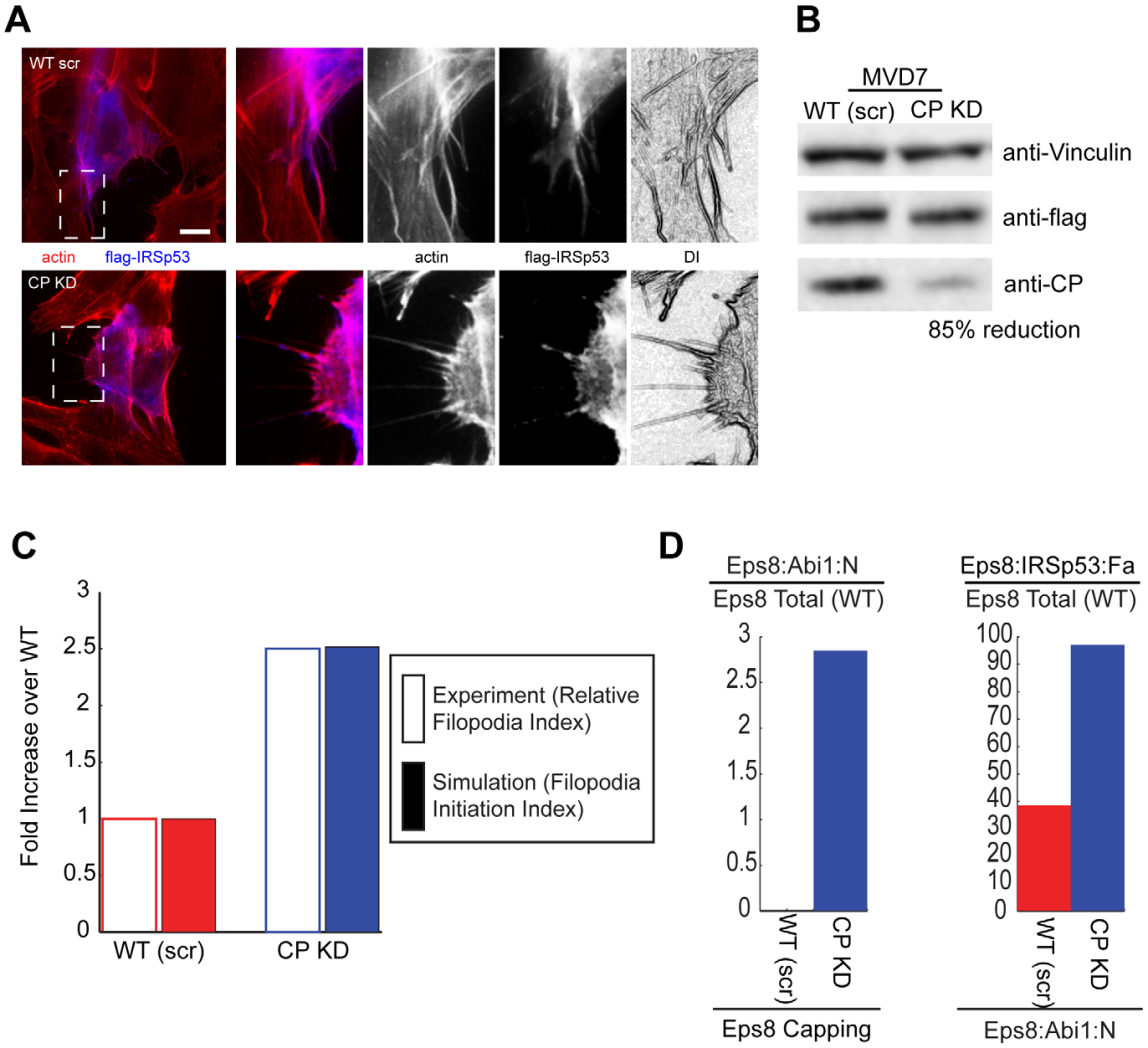
Eps8:IRSp53 induces filopodia formation in VASP-deficient MVD7 cells after RNAi-mediated removal of CP. **a.** RNAi-mediated downregulation of CP in MVD7 cells over-expressing IRSp53 increases filopodia formation. Control (WT scr) or CP (CP KD) RNAi-treated MVD7 cells transfected with Flag–IRSp53 were fixed and stained with rhodamine–phalloidine or anti-flag to detect F-actin (red) or IRSp53 (blue), respectively. Right panels, magnifications corresponding to the white dashed squares of the pictures on the left (the different channels are indicated). DI are digitalized images obtained with Adobe Photoshop filters starting from the actin channel to highlight cells protrusions [12]. Bar is 10 µm (4 µm for the magnifications). **b.** The expression of endogenous CP in cells interfered for CP (CP KD) or treated with scrambled oligo (WT scr) was analyzed by immunoblotting with the indicated abs. CP reduction (85%) was determined using the software ImageJ, by analyzing the intensity of the signals for CP in control cells (WT scr) or cells interfered for CP (CP KD), normalizing over vinculin signal. **c.** Change in *RFI* and *FII* (i.e., Eps8:IRSp53:Fa normalized by its wild type value, see main text) in WT and CP knockout MVD7 cells. Empty rectangles represent experimental results, filled rectangles simulations of equations in Table 1 in Text S1 and parameters in Table 2 and Table 3 in Text S1. **d.** Complexes formed in HeLa cells by Eps8, IRSp53 and Abi1 in CP Kd and WT plotted as percentage of total protein concentration in the wild type. Simulations as in **c**.

To test this prediction we analyzed the change in filopodia formation induced by CP removal in fibroblasts genetically devoid of VASP and MENA and expressing undetectable levels of EVL (MVD7 cells) [47]. We first measured the concentrations of IRSp53, Eps8 and Abi1, as compared to the concentrations measured in HeLa, and we found that MVD7 cells have less Abi1, more Eps8 and roughly the same concentration of IRSp53 (Fig. S2C and Table 2 in Text S1). Next, as these cells do not normally produce filopodia, we over-expressed IRSp53 (a condition called WT, in analogy to what done with HeLa cells) to induce these structures in a sizeable fraction of cells in the population, and we calculated the IRSp53-dependent relative filopodia index of CP knocked down cells with respect to scrambled siRNA-transfected cells (Fig. 6A–B). Using the calculated concentrations of the relevant proteins of MVD7 cells, while keeping the same binding parameters employed in HeLa (Table 2 in Text S1), the model predicted an increase of *FII* due to CP removal (Fig. 6C) as compared to the WT. The prediction was verified *in-vivo* by down-regulating CP via RNAi. Of note, the agreement between *FII* and *RFI* is quantitative.

According to the model, the increase in uncapped filaments leads to an increase in filamentous actin, and as a consequence to an increase in IRSp53:Eps8:Fa filopodia initiation complex (compare red and blue bars in Fig. 6D second panel). The increase in uncapped filaments also causes the amounts of Eps8:Abi-1 capping filaments to increase (compare red and blue bars in Fig. 6D first panel), but this was insufficient to compensate for the loss of CP due to the low amounts of Abi-1 present.

Our model predicts that a similar effect should also be observed in HeLa cells over-expressing IRSp53 (Fig. S3), where VASP is present but no longer capable of forming new initiation complexes since almost all VASP molecules are already present in VASP:IRSp53:Fa complexes (Fig. 4B third panel). Consistently, upon down-regulation of CP in HeLa cells via RNAi, we observed an increase in the measured filopodia index similar to that predicted *in-silico* (Fig. S3B). According to the model, CP removal does not increase the amount of VASP:IRSp53:Fa, already maximal, but increases both Eps8:Abi1:N and Eps8:IRSp53:Fa, this last having a stronger effect than the previous on filopodia formation (Fig. S3C).

We finally asked how much of these results were dependent on the precise choice of parameter values since, although most of them are experimentally measured, other parameters are not (Table 2 in Text S1). A sensitivity analysis showed that our results are largely independent on parameter values in both cell types (Fig. S4).

## Discussion

A number of proteins that regulate filopodia formation have multiple biochemically-diverse functions. For examples, VASP family proteins bundle filaments and protect barbed ends from cappers, Formins nucleate new linear filaments and protect barbed ends from capping, IRSp53 binds and bundles filaments and deforms the PM. Coherently, all those different roles act in concert to promote the formation of plasma membrane-linked, actin filament and bundles required to induce and/or sustain filopodia initiation or elongation. Eps8, instead, exerts actin-related biochemical roles that produce opposite biological effects (capping of filament ends that limits filament elongation, while crosslinking that promotes filament bundling) on filopodia formation. Our mathematical model shows that the dual function of Eps8 as a capper or bundler as function of the different Eps8 complexes can explain the seemingly paradoxical effects of Eps8 down-regulation in filopodia formation in different cell types. Thus we propose that Eps8 represents a molecular switch in the transduction of signaling, either directing the cells towards a reduction or an increase of filopodia, depending on the molecular context.

### Model's simplification

The model we propose is noticeably simple and yet it successfully reproduces experimental data and even predicts the outcome of new experiments. The biological system and experimental setting we employed justified some of the simplifications of the model. For example, since the phenotype we reproduce is a *RFI* that describes the time-averaged ability of cells to form filopodia, an overly detailed description of the physical process underlying filopodia formation is not required. For this reason, we did not take intra-cellular spatial localization into account and used values of total protein concentrations considering a cell as a well-stirred system.

Other simplifications concern the molecular players of our network. First, the model focuses only on a subset of well characterized molecules that are involved in generating filopodia, while it lacks some key components that have also recently been implicated in filopodia formation, such as formin or Myosin transporters, like Myosin X, or fascin. This choice was based on recent experiments that have revealed that multiple and independent mechanisms of filopodia formations may concomitantly operate. Indeed, it was recently shown that in neurons filopodia could be formed even after the ablation of all three VASP family members upon expression of Myosin X or the activated Formin, mDia2 [46]. This result was the basis to exclude the above-mentioned molecular pathways from our model.

Secondly, we neglected some components even within the pathway that we considered explicitly, as in the case of the cross linker fascin, which was shown to be essential for filopodia stabilization [8]. In this case, we hypothesize that diverse crosslinking proteins or protein complexes may all be required and act in a hierarchical and coordinated manner to promote filopodia formation. Under this scenario, complexes formed between filamentous actin, IRSp53 and its binding partners Eps8 or VASP may serve as the “initiators” of filopodia by promoting the “convergence and bundling” of actin filaments close to the barbed ends oriented toward the PM mainly by virtue of the established properties of IRSp53 to sense membrane curvature and promote convex membrane deformation. Such a mechanism for the initiation of membrane protrusions driven by actin polymerization was proposed from theoretical analysis [39], [41]. The good agreement found in our study between the experimental results and those obtained with our modeling supports this mechanism for filopodia initiation, and suggests that this hypothesis is worth further investigation.

Thirdly, we have not included all the known biological roles of the proteins under consideration. In particular, recent work showed that VASP may also act as an anti-capper and promote in a processive manner filament elongation [4], . Notably, these latter activities become significant mainly upon high-density clustering of VASP. Although we have not explicitly introduced these additional biochemical properties of VASP, they are partly intrinsic in our model as they may occur in later critical steps of filopodia extension. Our model, indeed, addresses what might be the very first step of filopodia fomation that requires the deformation of the plasma membrane and its coupling with the generation of actin filament bundles to support extension. This event is possibly initiated by proteins, such as IRSp53, and promoted, in a feedback loop fashion (as proposed in [39], [41]) by the bundling of pre-existing filaments. Within this context IRSp53 and VASP may act synergically (as a physically tethered complex) to cause filament bundling, and increased barbed-end polymerization, thus increasing the local protrusive force acting on the membrane. The membrane-curvature sensing domain of IRSp53 completes the positive feedback loop by causing IRSp53:VASP aggregation at the tips of emergent membrane deformations. For the filopodia to grow beyond this initiation stage, the actin filaments must remain uncapped to elongate in a processive fashion and further stabilized into tight bundles by actin cross-linkers, such as fascin.

VASP is likely essential also in this “second phase” by sliding from the side to filament tips. Here, upon clusterization possibly promoted by IRSp53-bound to the deformed plasma membrane, VASP may elongate actin filaments while protecting them from capping. According to this hypothesis, the initial recruitment of VASP from IRSp53 has a dual role – both as a filament crosslinker, bundling actin filaments into sufficiently stiff bundles, and recruiting VASP in close proximity to sites of membrane deformation.

Finally, The *RFI* represents the fraction of cells that develop filopodia, and thus the probability that cells with a certain genetic background can develop filopodia. The formation of filopodia has been proposed to be triggered by initiators of filopodia at the plasma membrane, by a positive feedback loop [39],[41]. Based on this model, we find that the probability of forming filopodia is linearly proportional to the average concentration of filopodia initiation complexes (*FII*), the molecular species that contain both F-actin and IRSp53. The model shows that the “probability of forming filopodia” becomes significant only above a threshold value of the filopodia initiator complexes, and it saturates as the amounts of filopodia initiator complexes increase further (Fig. 3B). Interestingly, our finding that real cells obey the above linear relationship suggests that the physiological amounts of initiators in the WT cells are kept close to the threshold value. In this regime, small changes to the concentration of these initiators can both easily induce a consistent increase or decrease of the probability of developing filopodia, thereby determining precisely the number of filopodia forming cells in a population. This sort of behavior is in agreement with previous observations [33] that indicate that cells are naturally positioned close to the filopodia-formation threshold.

### Eps8 is at the centre of a plastic network that controls filopodia formation

Collectively, our systems analysis and experimental results provide a cogent molecular and mathematical framework to account for how the multifunctional activities of the components of this network, with particular emphasis on Eps8 and VASP family of proteins, are controlled in different cellular context. The variable formation of distinct protein complexes either exerting capping activities or promoting filament bundling is key in determining the final biological output through quantitative relationships that only a systems biology approach could reveal. It is of note, for example, that the diverse combinatorial arrangement of a limited numbers of components ensures a level of unexpected plasticity of the network, so that seemingly opposite actin-related activities (from capping to anticapping and filament bundling) can be properly coordinated, ultimately differentially controlling the promotion of filopodia. One implication of these finding is that filopodia may not be considered entities governed by different and entirely independent molecular pathways [46]. Rather, the formation of these structures is finely regulated by a unique network connecting numerous molecular, presumably interchangeable and functionally redundant, players through distinct multi-protein complexes. In this context, our study shows clearly the potential of differentially expressing components of the network in terms of filopodia formation, as HeLa, MVD7 fibroblasts and neurons differ for the total concentration of 3 proteins of the network, and yet the effect in terms of filopodia is dramatic. The experiments performed in VASP-family-deficient MVD7 cells further show how the dynamic interplay of the components of the network underlying filopodia formation makes the system robust even to drastic changes, such as the absence of apparently essential components. In conclusion, our results suggest that the outer layer controlling filopodia formation plays a critical role to make the machinery controlling filopodia formation at the same time adaptable and capable of responding to different extracellular stimuli and environmental conditions.

## Materials and Methods

### Expression vectors, antibodies, reagents and cells

Cytomegalovirus (CMV)-promoter-based and elongation factor-1 (EF1) promoter-based eukaryotic expression vectors and GST bacterial expression vectors were generated by recombinant PCR. Myc–IRSp53 was a gift from S. Krugman (The Babraham Institute, Babraham Research Campus, Cambridge, UK). All constructs were sequence verified. The antibodies used were: monoclonal anti-Eps8 (Transduction Laboratories, Lexington, KY); rabbit polyclonal anti-GST, anti-Myc 9E10 (Babco, Berkeley, CA); anti-Flag M2 (Sigma-Aldrich, St Louis, MO); rabbit polyclonal anti-VASP (Immunoglobe, Himmelstald, Germany); monoclonal anti-Abi-1 was previously described [48] and monoclonal anti-IRSp53 [12]. HeLa knocked down for CP or control cells were obtained by transfecting cells with short hairpin loop oligos targeting human CP gene (AACCTCAGCGATCTGATCGAC) or scramble oligos (AACCTCAGCGATCTGATTGAC) respectively. For MVD7 cells, we used two Stealth RNAi oligos (Invitrogen) targeting murine CP (T1 = GAACCUCAGCGAUCUGAUCGACCUG; T2 = GAAGCACGCUGAAUGAGAUCUACUU) in combination with the appropriate scrambled oligo (scr T1s = GAACCUCAGUGAUCUGAUUGACCUG; scr T2s = AAGUAGAUUUCAUUAAGCGUGCUUC), as control.

### Protein purification

His–Eps8 FL and His-IRSp53 were obtained as previously described [12]. Recombinant VASP was expressed as GST fusion protein in the BL21 Escherichia colistrain(Stratagene, Cedar Creek, TX) and affinity purified using GS4B glutathione–Sepharose beads (Amersham Pharmacia Biotech, Piscataway, NJ). Eluted proteins were dialyzed in 50 mM Tris–HCl, 150 mM NaCl, 1 mM DTT and 20% glycerol. GST–VASP was cleaved from the GST using the PreScission protease (Amersham Pharmacia Biotech, Piscataway, NJ) according to the manufacturer's instructions. Actin was isolated from rabbit muscles and purified in the Ca–ATP–G-actin form by Sephadex G-200 chromatography in G buffer (5 mM Tris–Hcl at pH 7.8, 0.1 mM CaCl2, 0.2 mM ATP, 1 mM DTT and 0.01% NaN3).

### Fluorescence microscopy of actin bundling

Monomeric G-actin was polymerized as previously described [12]. F-actin was mixed with varying concentrations of recombinant and purified proteins (as described in the text) in F-buffer and incubated at room temperature for 30 min. Actin was then labeled with rhodamine–phalloidine and 0.1% DABCO and 0.1% methylcellulose were added to the mixture. The samples were mounted between a slide and a coverslip coated with poly-lysine and imaged by fluorescence microscopy.

### Transfection and immunofluorescence microscopy

HeLa cells, Cos7 cells and Hippocampal neurons were cultured as described in [10], [12] and [16], respectively. VASP-family deficient cells (MVD7) were a kind gift from F. Gertler and were cultured as described [47]. HeLa, Cos7 and MVD7 cells seeded on gelatin and were transfected with the indicated expression vectors using FuGene (Invitrogen, Carlsbad, CA), according to the manufacturer's instructions. After 24 h, cells were processed for epifluorescence or indirect immunofluorescence microscopy. Briefly, cells were fixed in 4% paraformaldehyde for 10 min, permeabilized in 0.1% Triton X-100 and 0.2% BSA for 10 min and then incubated with the primary antibody for 45 min, followed by incubation with the secondary antibody for 30 min. F-actin was detected by staining with rhodamine–phalloidine at a concentration of 6.7 U ml−1.

### CP knock down experiments

Hela: Epitope-tagged IRSp53 expressing or control cells seeded on gelatine were transfected with CP or control oligos using Oligofectamine (Invitrogen, Carlsbad, CA), according to the manufacturer's instructions.

MVD7: Epitope-tagged IRSp53 expressing or control cells seeded on gelatine were subjected to a double transfection protocol with CP or control oligos using Lipofectamine RNAiMAX (Invitrogen, Carlsbad, CA), according to the manufacturer's instructions.

### Generation of digitalized images

Images were obtained by applying the Adobe Photoshop filter ‘find edges’ to outline the cell contour. The average total length of protrusions per cell extending from the cell soma was calculated using ImageJ program in at least 30 different cells in triplicate experiments and expressed as fold increase with respect to the average total length of protrusions in control cells. Similarly, the number of branches per cell was manually counted and expressed as above.

### Biochemical assays

Standard procedures in vitro binding, cell lysis and coimmunoprecipitation were as previously described [12].

### Mathematical simulations

We solved the equations at steady state using XPP-AUTO or MATLAB.

For numerical analysis in MATLAB, we used the SBtoolbox2 [49]. To do this, we translated the set of equations in SBmodel files.

The steady state of the system was found using the SBsteadystate function, a function that numerically calculates the eigenvalues of the Jacobian matrix of the system.

Exploration of the parameter space in the model was carried out either manually using the SBtoolbox2, or through optimization algorithms found in the SBPD package [49].

## Supporting Information

Figure S1 **VASP synergizes with IRSp53 in bundling actin filaments and in promoting filopodia formation.** A. VASP and Eps8 bundle actin filaments with low efficiency. Fluorescence microscopy-based F-actin-bundling assay. F-actin (1 µM) was incubated with 1 µM BSA as control or with increasing concentrations of either Eps8 or VASP. Actin filaments labeled with rhodamine–phalloidin were imaged using a fluorescence microscope as described in Materials and Methods and [12]). Representative images of bundles filaments are shown. B. The addition of IRSp53 increases the bundling efficiency of Eps8 and VASP. VASP is a much stronger bundler than Eps8 when in complex with IRSp53. F-actin (1 µM) was incubated with 5 µM IRSp53 alone or with the indicated concentrations of either Eps8 or VASP. F-Actin was visualized as described above. The quantification of the bundling efficiency determined by measuring the number of bundles/field is shown in Fig. 2A. C. The concomitant expression of VASP and IRSp53 causes filopodia formation *in-vivo*. Cos-7 cells transfected with Flag–IRSp53 or GFP–VASP alone or in combination were fixed and processed for epifluorescence microscopy to visualize GFP–VASP (green) and stained with phalloidin or anti-Flag to detect F-actin (red) or IRSp53 (blue), respectively. The concomitant expression of VASP and IRSp53 increased membrane protrusions, which adopted the shape of long and highly branched extensions (indicated by arrows), where VASP and IRSp53 localized. The middle panels represent threefold magnifications of the areas indicated in the top panels. Filopodia induced by either IRSp53 alone or in combination with VASP are indicated by arrowheads. Representative examples of the indicated transfected cells are shown also as digitalized images to highlight the contour of cells (lower panels). A protrusive index was determined by measuring the total length and the number of branches of these protrusions as described in [12]. VASP and IRSp53 co-expressing cells displayed a 2-fold increase in length and 3.1-fold increase in the number of branched extension as compared with cells expressing only IRSp53 (not shown). The scale bar represents 10 µm.
(TIF)

Figure S2 **Protein expression in HeLa, neurons and MVD7 cells.** A. Similar expression levels of endogenous IRSp53, Eps8 and CP were found in HeLa and Hippocampal Neurons(Hip). B. We also determined the cytoplasmic concentration of VASP and IRSp53 in HeLa. Total cellular lysates of an increasing number of HeLa cells and increasing amounts of recombinant human VASP (lower panels), or His-tagged IRSp53 (upper panels), used as standards, were resolved by SDS-PAGE and immunoblotted with the indicated abs. The following criteria were used to estimate the concentrations of these proteins in neurons reported in Table 2 in Text S1: i) we used previously estimated average cell volumes for both HeLa and Neuronal cells [12], [42], [44]); ii) in the case of VASP family members, the levels of expression could not be estimated in neurons, where, however, high levels of Evl and Mena have been previously determined [45]. Absolute values for the concentration of Abi and Eps8 were previously calculated in [12]. Notice how, due to overexpression, in our simulations we use a higher value for the total concentration of IRSp53 in HeLa cells (i.e., total IRSp53 is the sum of the endogenous, reported here, and the overexpressed), Table 2 in Text S1. C) The levels of Eps8, Abi1 and IRSp53 in MVD7 and mouse embryo fibroblasts (MEFs) cells were measured as a fraction of their level of expression in HeLa cells.
(TIF)

Figure S3 **CP removal enhances IRSp53-mediated filopodia formation in HeLa cells.** A. RNAi-mediated downregulation of CP in HeLa cells over-expressing IRSp53 increases filopodia formation. Upper panels, control (WT scr) or CP (CP KD) RNAi-treated HeLa cells transfected with Myc–IRSp53 were fixed and stained with rhodamine–phalloidin or anti-myc to detect F-actin (red) or IRSp53 (green), respectively. Bar is 10 µm. Middle panels, images corresponding to the actin channel. Lower panels, digitalized images obtained with Adobe Photoshop filters to highlight cells protrusions [12]. The expression of Myc-IRSp53 and endogenous CP in interfered (CP KD) or control (WT scr) cells was analyzed by immunoblotting with the indicated abs. A reduction in CP levels after RNAi of about 85% was determined using the software ImageJ, by analyzing the intensity of the signals of protein bands corresponding to CP in control cells (WT scr) or CP knocked down cells (CP KD) after normalizing for the total amounts of proteins loaded in each lane with the vinculin signal. B. Change in RFI and FII (i.e., Eps8:IRSp53:Fa and VASP:IRSp53:Fa normalized by their wild type value, see main text) in WT and CP knocked down HeLa cells. Empty rectangles represent experimental results (see Table 3 in Text S1), filled rectangles simulations of equations in Table 1 in Text S1 and parameters in Table 2 and Table 3 in Text S1. C. Complexes formed in HeLa cells by Abi1, Eps8, IRSp53, and VASP in different genetic backgrounds plotted as percentage of total protein concentrations in the wild type. Simulations as in B.
(TIF)

Figure S4
**Sensitivity analysis.** To verify the robustness of the model described in the text, we measured the sensitivity coefficients σ, defined as 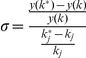 where y is the observable *FII* and j the parameter variation *k_j_*. *k* stands for a vector containing all the parameters of the model, and k^*^ stands for the same vector with *k_j_* substituted by k_j_^*^. For the three cellular types – HeLa (A), Neurons (B) and MVD7 cells (C) – we have considered the *FII* for the WT. We calculated the expression above using a custom MATLAB scripts (available upon request) by increasing or decreasing all the parameters in the model by plus or minus 1% (green and blue bars in the Figure, respectively). A change in the observable of over 1% indicates a sensitive parameter, while a change below 1% suggests that the model is robust to changes in that parameter. In the Figure, we plot σ as a function of all parameters in the wild type. Our simulations are largely independent on parameter values in the three cell types, with the exception of the ratio of the concentration of capping protein and the number of filament ends. Perturbing this ratio causes a significant change in the polymerization of actin: by slightly increasing the ratio of uncapped barbed ends, we observe a large increase in the amount of actin polymerized at steady state in our model. As discussed in the “Capping” section, this result is consistent with the fact that cells are exquisitely sensitive to the number of uncapped barbed ends.(TIF)

Text S1 **Contains equations and parameters used for the simulations.** Fig. S1 shows that both *in vivo* and *in vitro* VASP synergizes with IRSp53 in bundling actin filaments and in promoting filopodia formation. Fig. S2 reports the quantification of protein Expression in HeLa, Neurons and MVD7 cells. Fig. S3 shows that CP removal enhances IRSp53-mediated filopodia formation in HeLa cells. Fig. S4 shows the results of stability analysis of the model.
(DOC)
