# Supplementary figures and images for: The Eps8/IRSp53/VASP Network Differentially Controls Actin Capping and Bundling in Filopodia Formation
Source: PLoS Comput Biol. 2011 Jul 21;7(7):e1002088. doi: 10.1371/journal.pcbi.1002088 (PMC3140970; doi:10.1371/journal.pcbi.1002088)

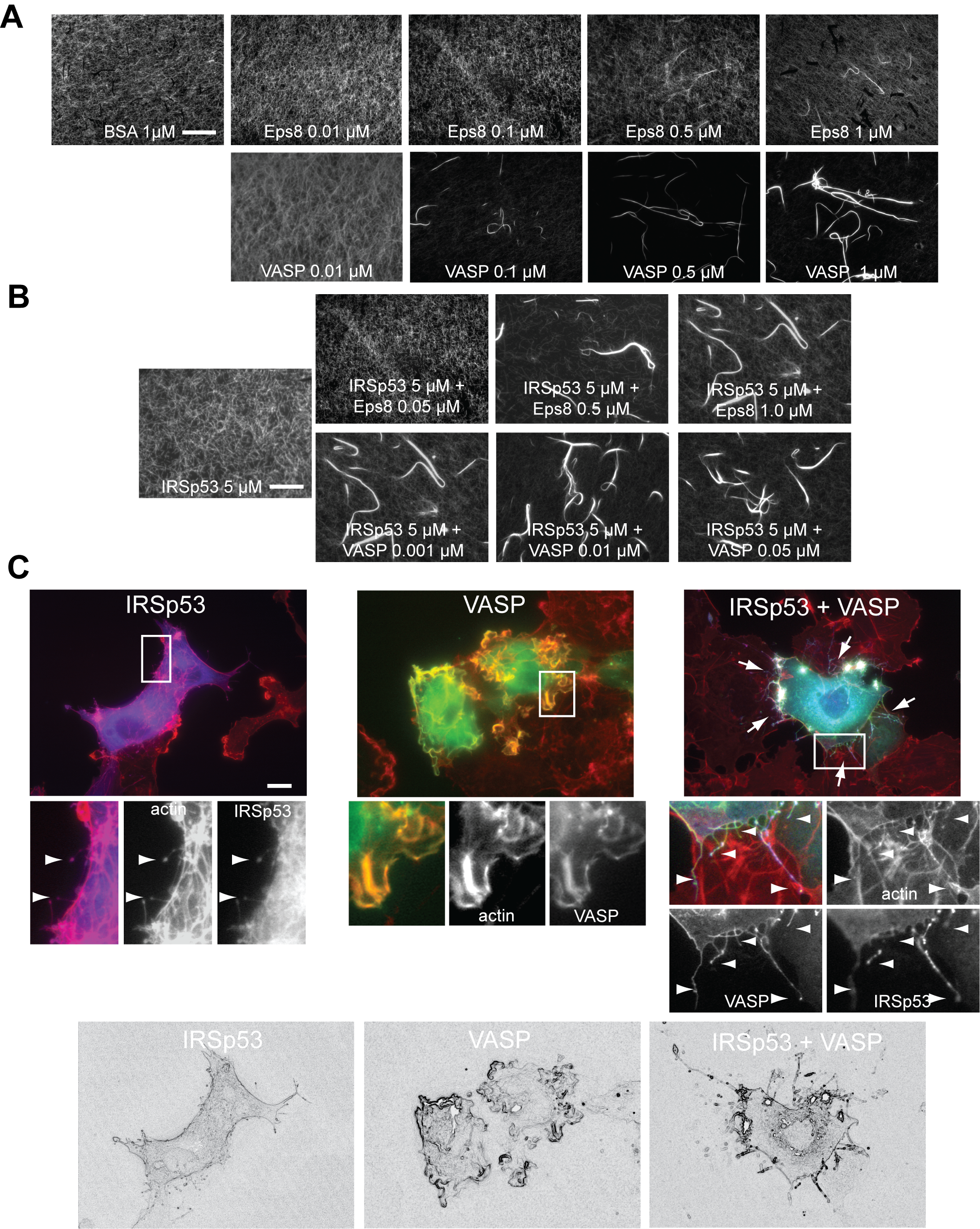

Supplement: Figure S1 — VASP synergizes with IRSp53 in bundling actin filaments and in promoting filopodia formation. A. VASP and Eps8 bundle actin filaments with low efficiency. Fluorescence microscopy-based F-actin-bundling assay. F-actin (1 µM) was incubated with 1 µM BSA as control or with increasing concentrations of either Eps8 or VASP. Actin filaments labeled with rhodamine–phalloidin were imaged using a fluorescence microscope as described in Materials and Methods and [12]). Representative images of bundles filaments are shown. B. The addition of IRSp53 increases the bundling efficiency of Eps8 and VASP. VASP is a much stronger bundler than Eps8 when in complex with IRSp53. F-actin (1 µM) was incubated with 5 µM IRSp53 alone or with the indicated concentrations of either Eps8 or VASP. F-Actin was visualized as described above. The quantification of the bundling efficiency determined by measuring the number of bundles/field is shown in Fig. 2A. C. The concomitant expression of VASP and IRSp53 causes filopodia formation in-vivo. Cos-7 cells transfected with Flag–IRSp53 or GFP–VASP alone or in combination were fixed and processed for epifluorescence microscopy to visualize GFP–VASP (green) and stained with phalloidin or anti-Flag to detect F-actin (red) or IRSp53 (blue), respectively. The concomitant expression of VASP and IRSp53 increased membrane protrusions, which adopted the shape of long and highly branched extensions (indicated by arrows), where VASP and IRSp53 localized. The middle panels represent threefold magnifications of the areas indicated in the top panels. Filopodia induced by either IRSp53 alone or in combination with VASP are indicated by arrowheads. Representative examples of the indicated transfected cells are shown also as digitalized images to highlight the contour of cells (lower panels). A protrusive index was determined by measuring the total length and the number of branches of these protrusions as described in [12]. VASP and IRSp53 co-expressing cells display [file pcbi.1002088.s001.tif]

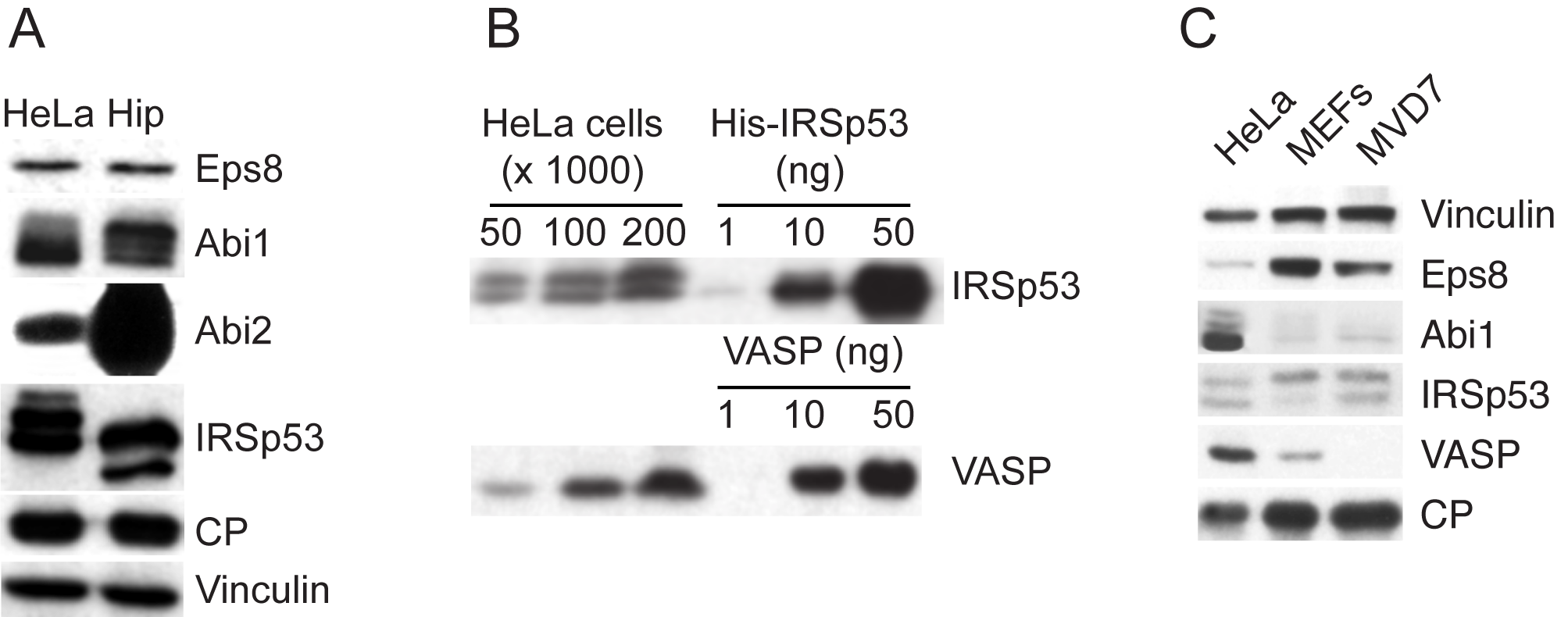

Supplement: Figure S2 — Protein expression in HeLa, neurons and MVD7 cells. A. Similar expression levels of endogenous IRSp53, Eps8 and CP were found in HeLa and Hippocampal Neurons(Hip). B. We also determined the cytoplasmic concentration of VASP and IRSp53 in HeLa. Total cellular lysates of an increasing number of HeLa cells and increasing amounts of recombinant human VASP (lower panels), or His-tagged IRSp53 (upper panels), used as standards, were resolved by SDS-PAGE and immunoblotted with the indicated abs. The following criteria were used to estimate the concentrations of these proteins in neurons reported in Table 2 in Text S1: i) we used previously estimated average cell volumes for both HeLa and Neuronal cells [12], [42], [44]); ii) in the case of VASP family members, the levels of expression could not be estimated in neurons, where, however, high levels of Evl and Mena have been previously determined [45]. Absolute values for the concentration of Abi and Eps8 were previously calculated in [12]. Notice how, due to overexpression, in our simulations we use a higher value for the total concentration of IRSp53 in HeLa cells (i.e., total IRSp53 is the sum of the endogenous, reported here, and the overexpressed), Table 2 in Text S1. C) The levels of Eps8, Abi1 and IRSp53 in MVD7 and mouse embryo fibroblasts (MEFs) cells were measured as a fraction of their level of expression in HeLa cells. (TIF) [file pcbi.1002088.s002.tif]

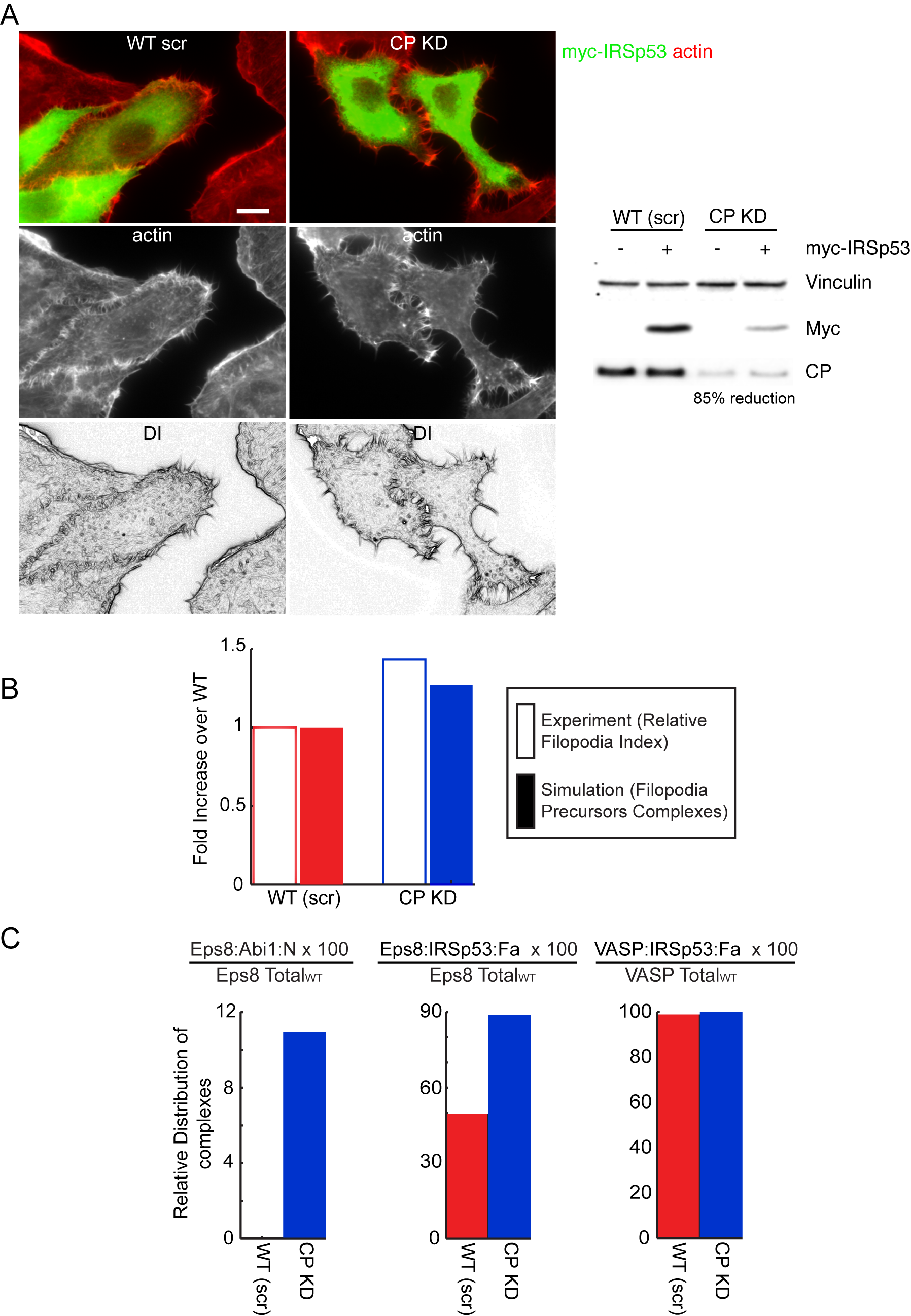

Supplement: Figure S3 — CP removal enhances IRSp53-mediated filopodia formation in HeLa cells. A. RNAi-mediated downregulation of CP in HeLa cells over-expressing IRSp53 increases filopodia formation. Upper panels, control (WT scr) or CP (CP KD) RNAi-treated HeLa cells transfected with Myc–IRSp53 were fixed and stained with rhodamine–phalloidin or anti-myc to detect F-actin (red) or IRSp53 (green), respectively. Bar is 10 µm. Middle panels, images corresponding to the actin channel. Lower panels, digitalized images obtained with Adobe Photoshop filters to highlight cells protrusions [12]. The expression of Myc-IRSp53 and endogenous CP in interfered (CP KD) or control (WT scr) cells was analyzed by immunoblotting with the indicated abs. A reduction in CP levels after RNAi of about 85% was determined using the software ImageJ, by analyzing the intensity of the signals of protein bands corresponding to CP in control cells (WT scr) or CP knocked down cells (CP KD) after normalizing for the total amounts of proteins loaded in each lane with the vinculin signal. B. Change in RFI and FII (i.e., Eps8:IRSp53:Fa and VASP:IRSp53:Fa normalized by their wild type value, see main text) in WT and CP knocked down HeLa cells. Empty rectangles represent experimental results (see Table 3 in Text S1), filled rectangles simulations of equations in Table 1 in Text S1 and parameters in Table 2 and Table 3 in Text S1. C. Complexes formed in HeLa cells by Abi1, Eps8, IRSp53, and VASP in different genetic backgrounds plotted as percentage of total protein concentrations in the wild type. Simulations as in B. (TIF) [file pcbi.1002088.s003.tif]

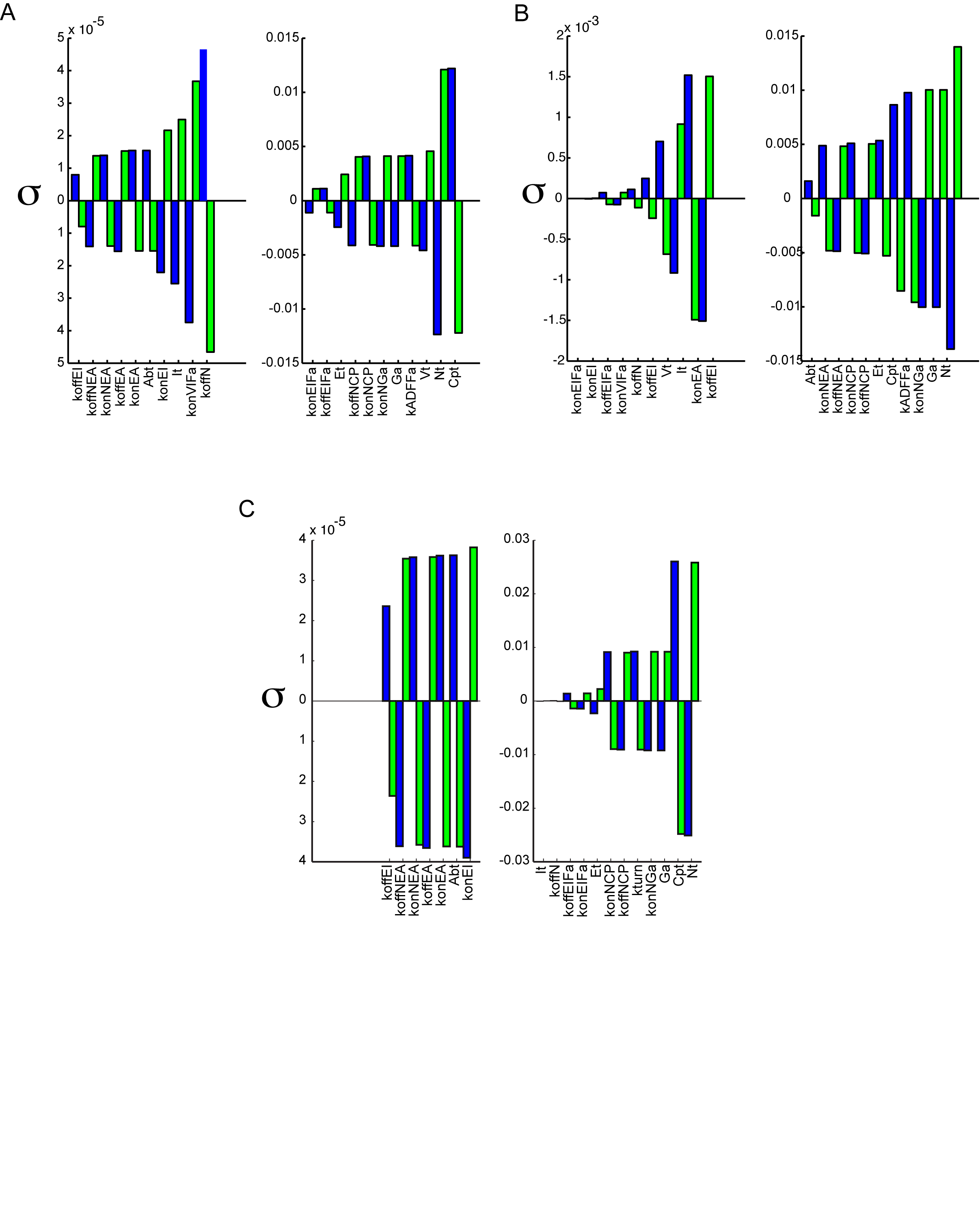

Supplement: Figure S4 — Sensitivity analysis. To verify the robustness of the model described in the text, we measured the sensitivity coefficients σ, defined as where y is the observable FII and j the parameter variation kj. k stands for a vector containing all the parameters of the model, and k* stands for the same vector with kj substituted by kj*. For the three cellular types – HeLa (A), Neurons (B) and MVD7 cells (C) – we have considered the FII for the WT. We calculated the expression above using a custom MATLAB scripts (available upon request) by increasing or decreasing all the parameters in the model by plus or minus 1% (green and blue bars in the Figure, respectively). A change in the observable of over 1% indicates a sensitive parameter, while a change below 1% suggests that the model is robust to changes in that parameter. In the Figure, we plot σ as a function of all parameters in the wild type. Our simulations are largely independent on parameter values in the three cell types, with the exception of the ratio of the concentration of capping protein and the number of filament ends. Perturbing this ratio causes a significant change in the polymerization of actin: by slightly increasing the ratio of uncapped barbed ends, we observe a large increase in the amount of actin polymerized at steady state in our model. As discussed in the “Capping” section, this result is consistent with the fact that cells are exquisitely sensitive to the number of uncapped barbed ends. (TIF) [file pcbi.1002088.s004.tif]
