# Supplementary material for: The Eps8/IRSp53/VASP Network Differentially Controls Actin Capping and Bundling in Filopodia Formation
Source: PLoS Comput Biol. 2011 Jul 21;7(7):e1002088. doi: 10.1371/journal.pcbi.1002088 (PMC3140970; doi:10.1371/journal.pcbi.1002088)
Supplement: Text S1 — Contains equations and parameters used for the simulations. Fig. S1 shows that both in vivo and in vitro VASP synergizes with IRSp53 in bundling actin filaments and in promoting filopodia formation. Fig. S2 reports the quantification of protein Expression in HeLa, Neurons and MVD7 cells. Fig. S3 shows that CP removal enhances IRSp53-mediated filopodia formation in HeLa cells. Fig. S4 shows the results of stability analysis of the model. (DOC) [file pcbi.1002088.s005.doc]

**Supplementary Material**

**Table 1- Algebraic-Differential Equations**

**Table 2 - Parameters used in the model**

**Table 3** **- List of phenotypes reproduced**

**Table 1- Algebraic-Differential Equations.**


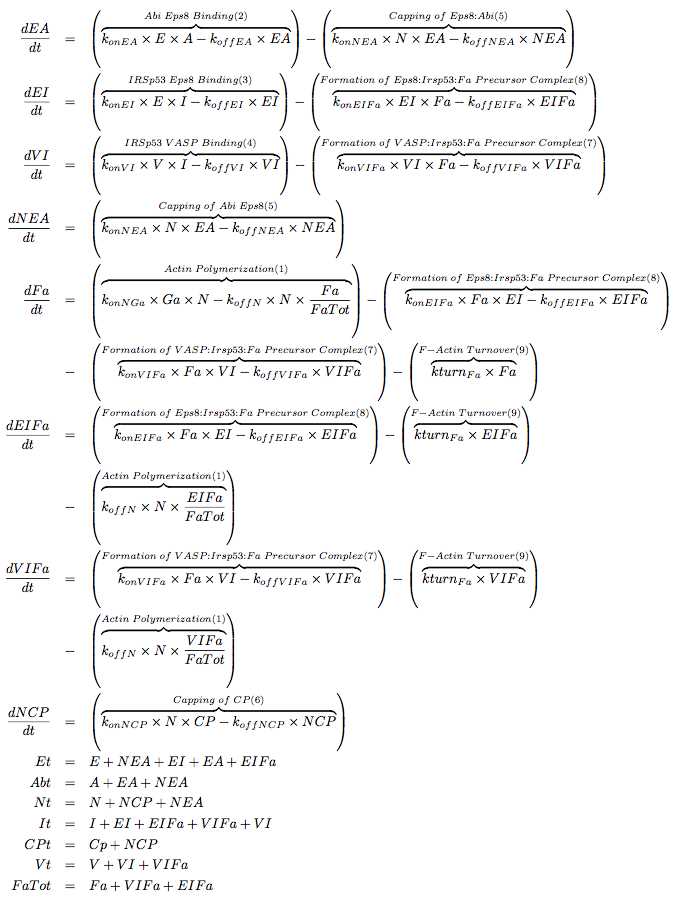


Ordinary differential equations used to translate Figure 1 into mathematical formalism. For each equation we give a description of the reactions that contribute to the right-hand side. Each reaction is characterized by a number, which can be used to identify the reactions in Figure 1. The names of the different chemical species can also be traced in Figure 1. We assumed mass-action kinetics for all reactions, see main-text for further details on derivation of all equations. The total amounts of Eps8 (Et), Abi1-2 (At), Irsp53 (It), VASP (Vt), Barbed Ends (Nt) and Capping Protein (CPt) are assumed to be constant. We verified through parameter search that the system is monostable.

**Table 2 - Parameters used in the model**

| **Reaction Rates** | | | | | |
| --- | --- | --- | --- | --- | --- |
| *Reaction Name* | *Forward Rate (μM^-1^ sec^-1^)* | | *Reverse Rate*  *(sec^-1^)* | *Reference* | |
| (1) Actin polymerization | k_onNGA_=11.6 | | k_offN_=1.3 | [1] | |
| (2) Abi Eps8 Binding | k_onEA_=1 | | k_offEA_=3 | [2] | |
| (3) IRSp53 Eps8 Binding | k_onEI_=1 | | k_offEI_=0.01 | [3] | |
| (4) IRSp53 VASP binding | k_onVI_=1 | | k_offVI_=0.01 | This paper (Fig. 2B) | |
| (5) Capping of Abi:Eps8 | k_onNEA_=210 | | k_offNEA_=0.08 | [2] | |
| (6) Capping of CP | k_onNCP_=6.3 | | k_offNCP_=0.0005 | [4] | |
| (7) Formation of VASP:Irsp53:Fa Initiation Complex | k_onVIFa_=100 | | k_offVIFa_=0.4 | This paper (estimated from Fig. 2a and S1a-b) | |
| (8) Formation of Eps8:Irsp53:Fa Initiation Complex | k_onEIFa_=1 | | k_offEIFa_=0.4 | [2,5] | |
| (9) F-Actin Turnover |  | | k_TURN_=0.01 | This paper (fitting) | |
| **Total Concentration of species** | | | | | |
| *Species* | | *Concentration in WT HeLa (μM)*  *Concentration in WT Neuron (μM)*  *Concentration in WT MVD7 (μM)* | | | *Reference* |
| Eps8 (Et) | | 0.83  0.83  3.6 | | | [2]  [6]  This paper (Fig. S2) |
| Barbed Ends (Nt) | | 0.2  0.2  0.2 | | | This paper (fitting) |
| IRSp53 (It) | | 12.6  1.2  13 | | | This paper (Fig. S2, overexpression as x10)  This paper (Fig. S2)  This paper (Fig. S2, overexpression as x10) |
| VASP (Vt) | | 0.86  3.5  0 | | | This paper (Fig.S2)  [7,8,9]  [10] |
| Abi-1/2 (Abt) | | 2  10  0.56 | | | [2]  This paper (Fig. S2)  This paper (Fig. S2) |
| G-Actin (Ga) | | 10  10  10 | | | [11]  [11]  [11] |
| Capping Protein (Cpt) | | 0.3  0.3  0.3 | | | This paper (fitting) |

Upper: we assume that the kinetics parameters governing the reaction rates are shared between WT HeLa, WT Neurons and WT MVD7 cells. The numbers that characterize the reactions are also shown in the network of Figure 1. The references cited provide an estimate for the dissociation constant of the reactions. Lower: The concentrations of the different species is the same or differ in the two cells types depending on the species, as reported in the Table and in the main text. Protein concentrations were either obtained from the literature, measured directly (see also Fig. S2) or obtained by parameter fitting. Notice that IRSp53 is overexpressed in HeLa and MVD7, and thus its value is increased compared to Neurons. Moreover, in neurons VASPt includes all VASP-family proteins and Abi-1/2t includes Abi2 as well, both very scarce in HeLa. As for the total concentration of F-actin in a cell, it was reported that the concentration of G-actin is roughly 3 times less than polymerized actin (F-Actin) [12]. However, only a minor fraction contributes to the formation of filopodia, while a large amount is found in structures, such as actomyosin filaments or stress fibers, not accessible to bundling proteins. Thus, we kept the F-actin concentration as an unconstrained variable, which was set after fitting to 2 µM.

**Table 3** **- List of phenotypes reproduced**.

| **Cell Type** | **Phenotype** | **Change in the Model** | **Reference** |
| --- | --- | --- | --- |
| HeLa | Eps8 Kd | Et=0.083 | [3] |
|  | VASP Kd | Vt=0.083 | [3] |
|  | VASP&Eps8 Kd | Et=Vt=0.083 | [3] |
|  | Abi Kd | Abt=0.2 | [3] |
|  | CP Kd | CPt=0.03 | This paper |
| Neurons | Eps8 Ko | Et=0 | [6] |
|  | VASP Kd& Eps8 Ko | Et=0, Vt=0.35 | [6] |
| MVD7 | CP Kd | CPt=0.03 | This paper |

The different phenotypes have been simulated using the wild type parameters, shown in Table S2, except for those reported here. We assume that a K.d. reduces the concentration of the protein to 10%, and K.O. reduces it to 0%. The experimental values for the relative filopodia index reported in Fig. 4A, 5B and S3B can be found in the references reported in the table.

**REFERENCES**

1. Pollard TD (1986) Rate constants for the reactions of ATP- and ADP-actin with the ends of actin filaments. J Cell Biol 103: 2747-2754.

2. Disanza A, Carlier MF, Stradal TE, Didry D, Frittoli E, et al. (2004) Eps8 controls actin-based motility by capping the barbed ends of actin filaments. Nat Cell Biol 6: 1180-1188.

3. Disanza A, Mantoani S, Hertzog M, Gerboth S, Frittoli E, et al. (2006) Regulation of cell shape by Cdc42 is mediated by the synergic actin-bundling activity of the Eps8-IRSp53 complex. Nat Cell Biol 8: 1337-1347.

4. Wear MA, Yamashita A, Kim K, Maeda Y, Cooper JA (2003) How capping protein binds the barbed end of the actin filament. Curr Biol 13: 1531-1537.

5. Yamagishi A, Masuda M, Ohki T, Onishi H, Mochizuki N (2004) A novel actin bundling/filopodium-forming domain conserved in insulin receptor tyrosine kinase substrate p53 and missing in metastasis protein. J Biol Chem 279: 14929-14936.

6. Menna E, Disanza A, Cagnoli C, Schenk U, Gelsomino G, et al. (2009) Eps8 regulates axonal filopodia in hippocampal neurons in response to brain-derived neurotrophic factor (BDNF). PLoS Biol 7: e1000138.

7. Gambaryan S, Hauser W, Kobsar A, Glazova M, Walter U (2001) Distribution, cellular localization, and postnatal development of VASP and Mena expression in mouse tissues. Histochem Cell Biol 116: 535-543.

8. Lanier LM, Gates MA, Witke W, Menzies AS, Wehman AM, et al. (1999) Mena is required for neurulation and commissure formation. Neuron 22: 313-325.

9. Laurent V, Loisel TP, Harbeck B, Wehman A, Grobe L, et al. (1999) Role of proteins of the Ena/VASP family in actin-based motility of Listeria monocytogenes. J Cell Biol 144: 1245-1258.

10. Bear JE, Loureiro JJ, Libova I, Fassler R, Wehland J, et al. (2000) Negative regulation of fibroblast motility by Ena/VASP proteins. Cell 101: 717-728.

11. Novak IL, Slepchenko BM, Mogilner A (2008) Quantitative analysis of G-actin transport in motile cells. Biophys J 95: 1627-1638.

12. Abraham VC, Krishnamurthi V, Taylor DL, Lanni F (1999) The actin-based nanomachine at the leading edge of migrating cells. Biophys J 77: 1721-1732.
